# Predicting In-Hospital Mortality in Intensive Care Unit Patients Using Causal SurvivalNet With Serum Chloride and Other Causal Factors: Cross-Country Study

**DOI:** 10.2196/70118

**Published:** 2025-07-24

**Authors:** Jing Wang, Qixiu Li, Can Xie, Xiaofei Li, Huikao Wang, Wei Xu, Ruyan Lv, Xiaobing Zhai, Ping Xu, Kefeng Li, Xi-Cheng Song

**Affiliations:** 1 Department of Critical Care Medicine Yantai Yuhuangding Hospital Qingdao University Yantai China; 2 Faculty of Applied Science Macao Polytechnic University Macao Macao; 3 Emergency Department Zigong Fourth People’s Hospital Zigong China; 4 Department of Otolaryngology Head and Neck Surgery Yantai Yuhuangding Hospital Qingdao University Yantai China

**Keywords:** electrolyte imbalance, prognosis, deep learning, threshold, Causal SurvivalNet, intensive care unit, ICU

## Abstract

**Background:**

Incorporating initial serum chloride levels as a prognostic indicator in the intensive care environment has the potential to refine risk stratification and tailor treatment strategies, leading to more efficient use of clinical resources and improved patient outcomes.

**Objective:**

Quantitative analysis of the relationship between serum chloride levels at intensive care unit (ICU) admission and in-hospital mortality, and the establishment of a personalized survival curve prediction deep learning model to enhance risk stratification and clinical decision-making.

**Methods:**

A large-scale, cross-country, multicohort study of 189,462 ICU patients from four cohorts was conducted: 70,370 from Medical Information Mart for Intensive Care IV (MIMIC-IV), 112,457 from eICU Collaborative Research Database (eICU-CRD; 2 US cohorts), 4653 from Yantai Yuhuangding Hospital, and 1982 patients from Zigong Fourth People’s Hospital (2 Chinese cohorts). We collected demographics, underlying diseases, ICU complications, electrolyte levels, biochemical parameters, and vital signs at ICU admission, along with length of stay and in-hospital survival outcomes. Causal graph analysis pinpointed clinical variables linked to mortality. Nonlinear associations between chloride levels and mortality were evaluated using restricted cubic splines and Cox proportional hazards models, validated with the Cox frailty model, Kaplan-Meier curves, and sensitivity analyses. A deep learning model was created for individualized survival predictions.

**Results:**

Causal inference revealed a significant association between admission serum chloride levels and 28-day mortality. The median serum chloride level at ICU admission was 104 (IQR 100-108) mEq/L. In analyzing all 42 variables, restricted cubic splines identified thresholds at 103 mEq/L and 115 mEq/L, categorizing patients into three groups: ≤103 mEq/L, 103-115 mEq/L, and >115 mEq/L. Cox proportional hazards models revealed higher death risks for patients outside this range, with hazard ratios (HRs) of 1.36 (95% CI 1.29-1.43) for ≤103 mEq/L and 1.27 (95% CI 1.14-1.41) for >115 mEq/L. Four cross-cohort validations confirmed these critical ranges. For the eICU-CRD dataset, the HRs for the key intervals are 1.30 (95% CI 1.24-1.36) and 0.97 (95% CI 0.89-1.06). In the Yantai Yuhuangding Hospital affiliated with Qingdao University (YHD-HOSP) dataset, the HRs for the key intervals are 1.23 (95% CI 1.09-1.38) and 1.58 (95% CI 1.27-1.96). In the Sichuan Zigong Fourth People’s Hospital (SCZG-HOSP) dataset, the HR for the key interval is 2.20 (95% CI 1.43-3.39). The Causal SurvivalNet accurately predicted individual survival curves using admission chloride levels and other factors, achieving Brier scores of 0.09, 0.12, and 0.15. Results from cohort analyses in both China and the United States consistently and closely correlate the critical range of chloride with the prognosis of ICU patients.

**Conclusions:**

Using initial serum chloride levels enhances prognostic accuracy and facilitates tailored treatment plans for ICU patients in critical care settings.

## Introduction

Chloride ions, as the predominant anions in the human extracellular fluid, play a pivotal role in regulating a wide array of physiological functions, including osmotic balance, acid-base equilibrium, muscle function, and fluid regulation [1,2]. Abnormal serum chloride concentrations have been linked to the development of various clinical complications and an increased risk of mortality among intensive care unit (ICU) patients [3]. Despite its vital role, chloride often remains underappreciated in critical care medicine, with its significance often overshadowed by other electrolytes such as sodium and potassium.

Early studies have investigated the relationship between serum chloride levels at admission and patient mortality, suggesting a potential risk of inpatient death associated with abnormal chloride concentrations [4-6]. However, these investigations have primarily been conducted in small cohorts or specific disease populations. In addition, the majority of previous studies have used the reference normal ranges for chloride levels in the general population (96-108 mEq/L) [7]. This approach fails to account for the complexities of the critically ill patients, who may exhibit different chloride thresholds related to mortality. There is thus a pressing need for more specialized research to explore the association between serum chloride and mortality in broader ICU patient cohorts.

At present, the physiological significance of chloride levels in critically ill patients remains poorly understood, and traditional prognostic models primarily focus on analyzing correlations between clinical variables and outcomes. However, the introduction of causal inference methods offers a novel approach to addressing this issue. In recent years, with the increasing application of causal inference in medical research, its latest advancements provide robust support for further exploration of this problem. In medical research, causal inference involves concluding the causal relationships between variables based on observed data, typically using statistical methods to distinguish between mere associations and genuine causal effects. Advances in causal inference have introduced new statistical methods, such as the fast causal inference (FCI) technique [8]. This method was effectively applied in a recent study to investigate causal factors affecting hospital stay durations following cardiac surgery [9]. Nevertheless, the causal underpinnings of in-hospital mortality among ICU patients remain largely unexplored.

Conventional survival analysis approaches, such as the Cox proportional hazards (CPHs) model, often assume a uniform mortality risk across participants, which is implausible in the dynamic and nonlinear milieu of critical illness [10,11]. Moreover, these methods fall short of personalized survival predictions. In contrast, deep learning models, with their deep neural networks, can reveal hidden features and nonlinear relationships within the data [12,13]. This capability makes deep learning an appealing tool for exploring the complexities of critical care outcomes. Various deep learning architectures, including random survival forest, Cox-Time, and DeepHit, have been explored in the context of predicting patient survival [14,15]. Despite the promise shown by Cox-Time in cancer settings, its applicability and performance in intensive care contexts warrant further rigorous evaluation. In addition, enhancing the interpretability of these models is crucial for clinical use.

In this cross-country, large-scale cohort study, we aimed to quantitatively define the range of admissible serum chloride levels causally linked to high mortality rates in critically ill patients from diverse racial and social backgrounds. Our second objective was to develop a deep learning model for generating personalized survival curves for critically ill patients during hospitalization, incorporating serum chloride and other causal clinical variables associated with mortality, thus addressing the limitations of traditional methods. Finally, to enhance clinical applicability, we developed a user-friendly web tool named Causal SurvivalNet for our deep learning model.

## Methods

### Study Design and Setting

This large-scale, multicenter prospective longitudinal cohort study used four datasets: two from the United States—Medical Information Mart for Intensive Care IV (MIMIC-IV; 2008-2019) and eICU Collaborative Research Database (eICU-CRD; 2014-2015)—and two from China—Yantai Yuhuangding Hospital affiliated with Qingdao University (YHD-HOSP; 2021-2023) and Sichuan Zigong Fourth People’s Hospital (SCZG-HOSP; December 2016 to June 2019).

**Figure 1:**
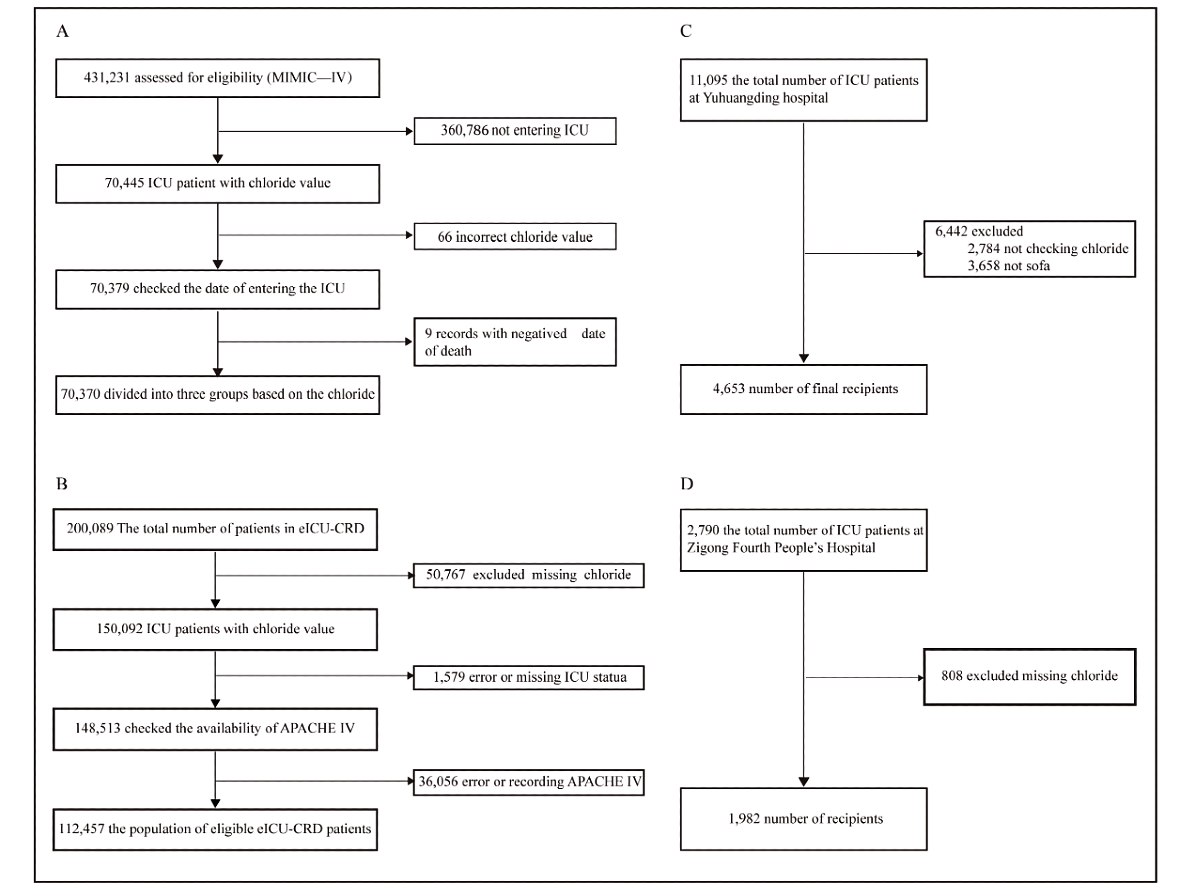
Patient selection flowcharts for four international cohorts used in this study: (A) MIMIC-IV (N=70,370), (B) eICU-CRD (N=112,457), (C) Yantai Yuhuangding Hospital affiliated with Qingdao University (N=4653), and (D) Sichuan Zigong Fourth People’s Hospital (N=1982). Detailed inclusion and exclusion criteria are provided in eText 1 in Multimedia Appendix 1. APACHE IV: Acute Physiology and Chronic Health Evaluation IV; eICU-CRD: eICU Collaborative Research Database; ICU: intensive care unit; MIMIC-IV: Medical Information Mart for Intensive Care IV.

Detailed inclusion criteria and selection strategies are outlined in Figure 1 and Text S1 in Multimedia Appendix 1, which describes the multidatabase ICU patient sample selection process, including inclusion and exclusion criteria. These measures were implemented to ensure sample representativeness and data quality, thereby establishing a foundation for subsequent clinical research on the association between serum chloride levels and in-hospital mortality. This work ultimately provides scientific evidence for ICU patient prognosis assessment and personalized treatment planning. In brief, we included patients who were admitted to the ICU with a valid serum chloride concentration recorded upon ICU admission. We excluded patients who were not admitted to the ICU, lacked chloride records, did not have Sequential Organ Failure Assessment (SOFA) or Acute Physiology and Chronic Health Evaluation IV (APACHE IV) scores, had negative ICU admission times, or had more than 20% of missing variable values. Duplicate records were removed, and each patient was retained with a single valid observation. Probabilistic principal component analysis was used to impute continuous variables with missing values <20%. Overall, the final cohort contains a total of 189,462 patients, including 70,370 patients from MIMIC-IV, 112,457 patients from eICU-CRD, 4653 patients from YHD-HOSP, and 1982 patients from SCZG-HOSP.

### Procedures

We conducted SQL queries on the MIMIC-IV, eICU-CRD, and SCZG-HOSP databases and accessed medical records from YHD-HOSP to extract clinical data and laboratory test results for all patients who were critically ill. The primary variable of interest was the initial serum chloride value recorded upon ICU admission. Covariates were categorized into six groups: demographic data, comorbidities, critical illnesses, ions and electrolytes, biochemical markers, and vital signs (Table S1 in Multimedia Appendix 1). The study aimed to investigate the association between these variables and in-hospital mortality. We extracted the first recorded laboratory test results and vital signs obtained at the time of ICU admission. SOFA or APACHE IV scores were included in the analysis whenever available (SOFA scores from MIMIC-IV and YHD-HOSP; APACHE IV scores from eICU-CRD; no scores from SCZG-HOSP) [16-19]. Disease classification used the *International Classification of Diseases and Related Health Problems, Tenth Revision (ICD-10*) codes provided by the World Health Organization (WHO) to minimize calculation errors and enhance the accuracy of disease classification [20].

### Outcomes

The primary outcome was 28-day in-hospital mortality (died or survived).

### General Statistical Analysis

In terms of general statistical analysis, continuous variables are depicted as median and IQR, while categorical variables are described as counts and proportions. Statistical analyses were performed using SPSS Statistics version 27 (IBM Corp) or R (version 4.4.0; R Core Team), unless otherwise specified.

### Causal Graphical Analysis

The FCI algorithm has demonstrated substantial value in medical research, achieving notable successes. A representative study on postoperative hospitalization used FCI-derived partial ancestral graphs to establish causal relationships between operative duration, preoperative comorbidities, and extended hospital stays, yielding actionable insights for clinical decision-making [9]. To investigate causal relationships among exposures, covariates, and outcomes, we used the FCI algorithm implemented in Tetrad visual software (version 7.5.0; Carnegie Mellon University) [21]. This method identifies conditional independencies within patient data to infer causal directions between variables and generate partial ancestral graphs [22]. For detailed procedures regarding the FCI algorithm, please refer to Text S2 in Multimedia Appendix 1. This algorithm provides a basis for causal inference in studying the impact of serum chloride levels on the prognosis of patients who were critically ill. It helps more accurately identify clinical variables associated with mortality, thereby optimizing risk assessment and guiding treatment decisions for ICU patients.

### Restricted Cubic Spline Analysis

The restricted cubic spline (RCS) analysis explored the nonlinear relationship between serum chloride levels and in-hospital mortality, identifying the critical ranges of chloride in ICU patients. The critical interval values were determined by identifying the points where the RCS curve intersects the reference line (hazard ratio, HR=1).

### Cox Regression and Cox Frailty Analysis Based on the Identified Crucial Ranges of Serum Chloride Level

Cox regression analysis was conducted to examine the association between serum chloride levels and in-hospital mortality (Text S3 in Multimedia Appendix 1), which quantified the relationship in patients who were critically ill to provide a statistical basis for developing prognostic models and individualized treatment strategies. To account for heterogeneity across ICUs and validate the Cox regression results, we applied the Cox frailty model, which introduces a frailty term to capture unobserved heterogeneity and thereby improves survival analysis accuracy (Text S4 in Multimedia Appendix 1) [23].

**Figure 2:**
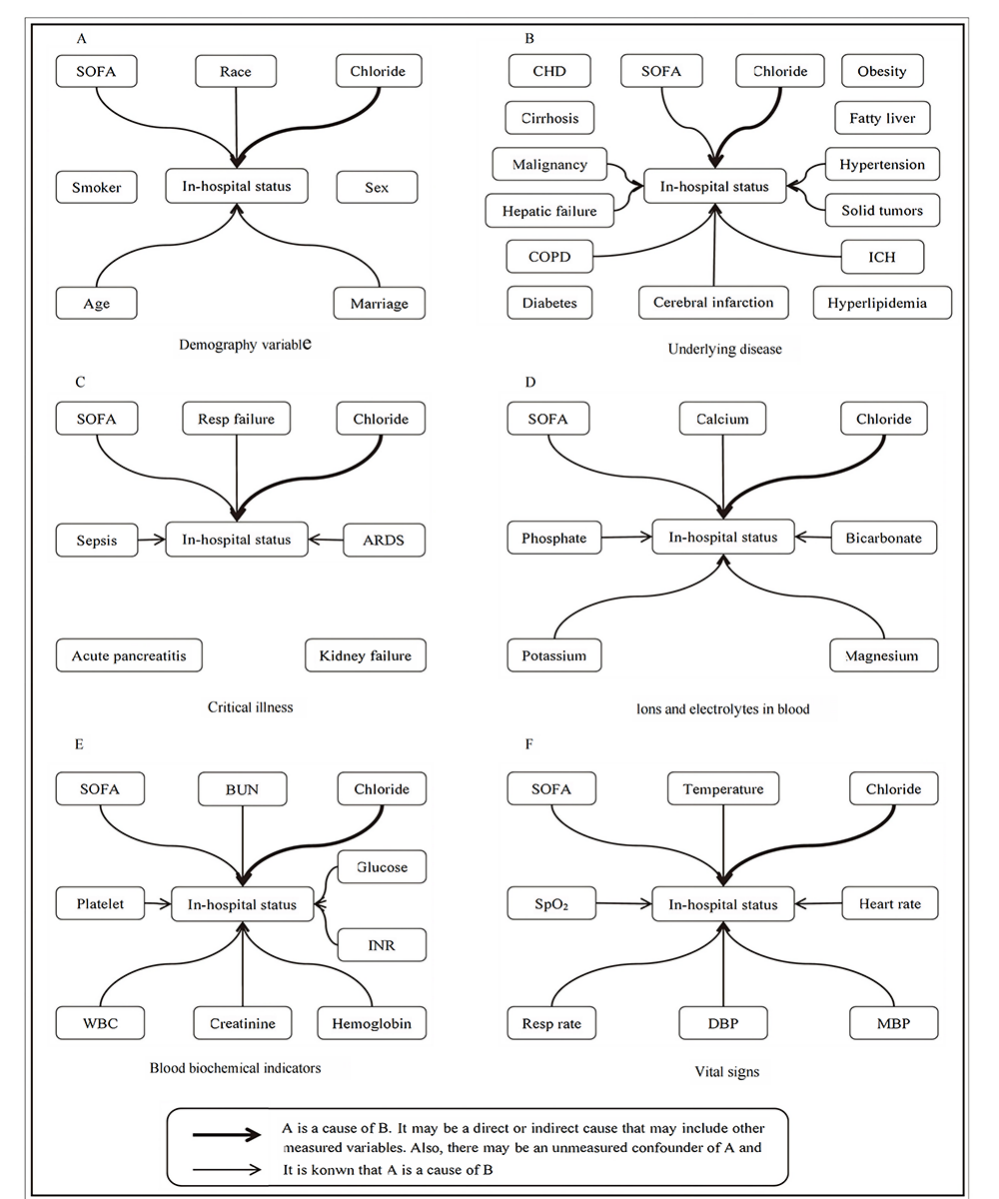
Causal analysis of clinical variables associated with in-hospital mortality based on Medical Information Mart for Intensive Care IV (MIMIC-IV) data: (A) demographic variables: race, (B) underlying diseases, (C) critical illnesses, (D) blood ions and electrolytes, (E) blood biochemical indicators, and (F) vital signs. Arrows denote potential causal links and their directions, with line thickness indicating the strength of these associations. Chloride, the primary exposure of interest, is prominently featured within the causal network. ARDS: acute respiratory distress syndrome; BUN: blood urea nitrogen; CHD: coronary heart disease; COPD: chronic obstructive pulmonary disease; DBP: diastolic blood pressure; ICH: intracerebral hemorrhage; INR: international normalized ratio; MBP: mean blood pressure; SOFA: Sequential Organ Failure Assessment; SpO2: peripheral oxygen saturation; WBC: white blood count.

Four models were constructed to assess the impact of different covariates:

- Model 0: unadjusted model, including only chloride and outcome variables.
- Model 1: built on Model 0 and included demographic information (age, sex, smoking status, marital status, and race), underlying diseases (chronic obstructive pulmonary disease, cirrhosis, hepatic failure, fatty liver, malignancy, cerebral infarction, coronary heart disease, solid tumors, intracerebral hemorrhage, hypertension, hyperlipidemia, diabetes, and obesity), complications (sepsis, acute pancreatitis, acute respiratory distress syndrome, kidney failure, and respiratory failure), and the SOFA and APACHE IV scores.
- Model 2: included the covariates from model 1 and incorporated ions and electrolytes in blood (potassium, phosphate, magnesium, and bicarbonate).
- Model 3: further extended model 2 by adding biochemical variables (platelet count, white blood cell count, creatinine, international normalized ratio, hemoglobin, glucose, and urea nitrogen) and vital sign variables (peripheral oxygen saturation, respiratory rate, mean blood pressure, diastolic blood pressure, heart rate, and temperature).
- Model 4: encompassed all causal variables identified through causal inference analysis (Figure 2).

The traditional Cox regression was implemented using IBM SPSS Statistics version 27, while the Cox frailty model was performed using the survival package (version 3.5-5) developed by Therneau et al [23] in R.

### Population-Based Survival Curves

We generated prognostic survival curves stratified by serum chloride intervals using the Kaplan-Meier method. This analysis was implemented in R (version 4.4.0), using the survival (version 3.5-5) package.

### Cross-Country and Multicohort Validation

We used the MIMIC-IV dataset from the United States as the discovery set, while datasets from the multicenter eICU-CRD in the United States and YHD-HOSP and SCZG-HOSP in China were used as validation sets. Initially, we divided the populations from eICU-CRD, YHD-HOSP, and SCZG-HOSP based on 3 chloride critical intervals calculated using MIMIC-IV. Afterwards, we used Cox regression and the Cox frailty model to calculate the HRs within these serum chloride intervals for the validation cohort. These HRs were compared with those from the discovery cohort to assess the reproducibility of the identified serum chloride thresholds associated with mortality. In addition, Kaplan-Meier survival curves stratified by serum chloride intervals were generated for the validation cohorts and compared with those from the discovery cohort to determine the consistency of survival probability patterns (Figure S1 in Multimedia Appendix 1). This approach systematically presents the complete research process—from data selection to causal analysis, model development, and multinational validation—offering clinicians an analytical framework based on serum chloride levels and other variables to identify key determinants of ICU in-hospital mortality and verify their generalizability.

### Sensitivity Analyses

The kidneys play a crucial role in regulating chloride homeostasis, as the reabsorption of chloride by renal tubules is essential for maintaining internal environmental stability [24]. Previous studies have identified a correlation between fluctuations in chloride levels and the occurrence of acute kidney injury (AKI) in patients [25]. Therefore, a sensitivity analysis was conducted to examine the impact of AKI on the association between serum chloride level and in-hospital mortality.

Sodium and chloride ions are physiologically interdependent and essential for critical functions. In critically ill patients, normal saline (NaCl solution) plays a vital role in fluid resuscitation and maintenance [26]. For those with chronic renal failure, precise regulation of sodium and chloride balance, through hemodialysis or other methods, is necessary to maintain electrolyte stability [27]. Given its clinical significance, sodium was not only included in our sensitivity analyses but also rigorously defined by specific concentration ranges (Figure S2 in Multimedia Appendix 1). This RCS curve analysis revealed a nonlinear relationship between sodium levels and in-hospital mortality in ICU patients. Combined with the chloride-level findings, these results establish clear risk thresholds for both key electrolytes in critically ill patients, providing clinicians with precise quantitative criteria to facilitate early identification of high-risk patients and optimization of electrolyte management strategies.

### Deep Learning–Based Personalized Survival Curves

This study combines artificial neural networks with the traditional CPH model to extract features from multidimensional patient data, optimize training parameters, and predict individual patient survival rates effectively [28]. The model was developed using PyCox (version 0.2.3; Joseph Ramsey et al) on the Anaconda platform (version 3; Peter Wang et al), the PyTorch framework (version 1.12.1; Soumith Chintala et al), and the programming language Python (version 3.9; Guido van Rossum) as described by Kvamme and Borgan [28]. The traditional CPH model (Text S5 in Multimedia Appendix 1) is constrained by its inherent linear assumptions, which can limit its learnability from complex, multidimensional patient data.

**Figure 3:**
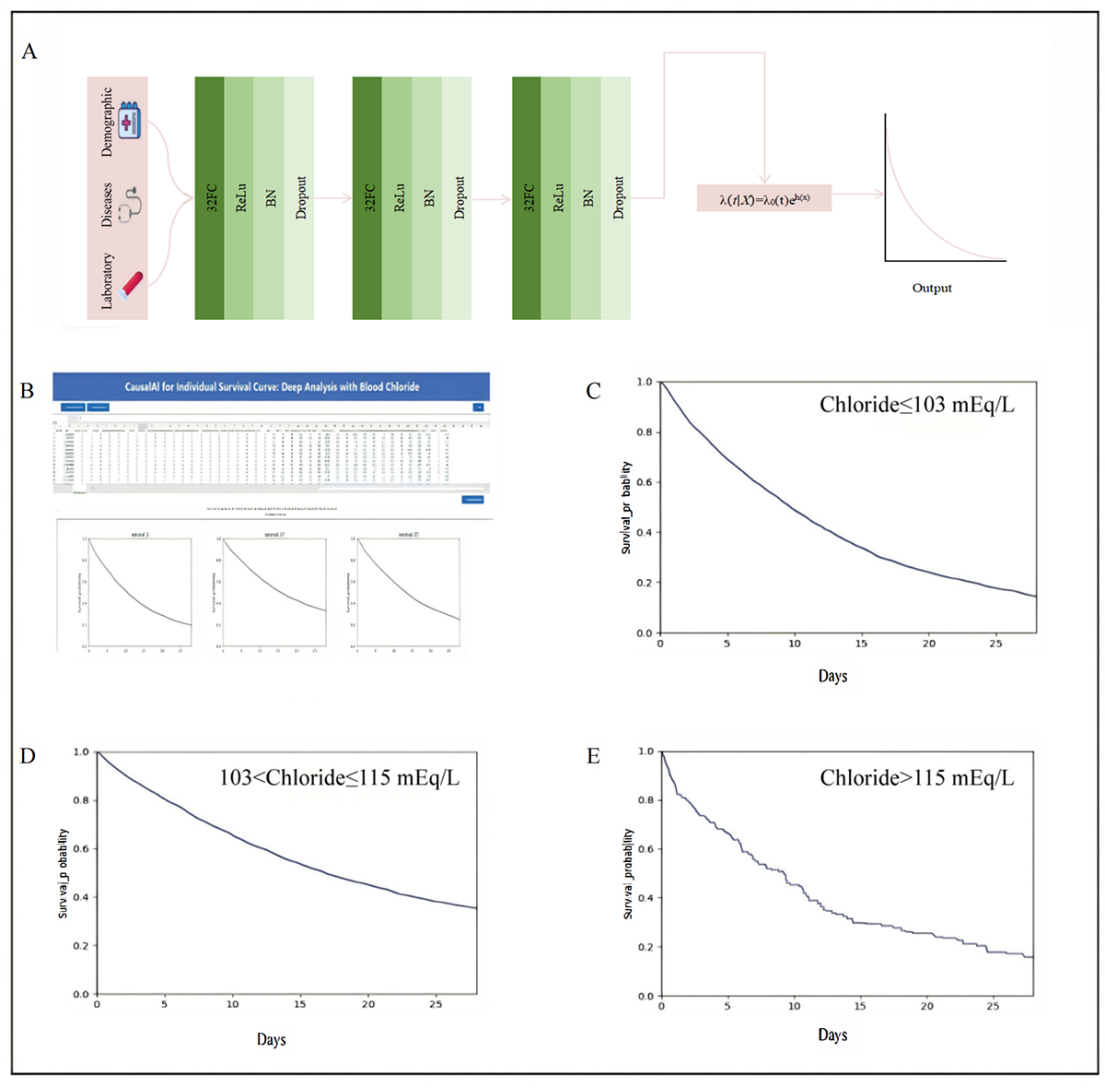
Modeling personalized survival curves and mortality prediction for intensive care unit patients using Causal SurvivalNet: (A) schematic of the Causal SurvivalNet deep learning model, (B) a screenshot of the web-based tool developed from the Causal SurvivalNet, and (C-E) representative personalized 28-day survival curves for intensive care unit patients, stratified by serum chloride levels. BN: batch normalization; ReLu: rectified linear unit.

To overcome these limitations, we propose integrating trainable neural network weights through fully connected layers into the CPH framework (Text S6 in Multimedia Appendix 1). This approach maintains the fundamental units of the CPH model while introducing neural network layers for enhanced modeling capability. To further improve the model’s nonlinearity, we incorporate an additional activation function layer after the fully connected (FC) layer, as illustrated in Figure 3A.

The learning process of the deep survival analysis algorithm (Text S7 in Multimedia Appendix 1) involves updating weights. The deep survival analysis algorithm performs weight updates using learning rate and L2 regularization derivatives. We enhanced the architecture by adding ReLU activation after the fully connected layer to simplify gradient computation and batch normalization to standardize input data distribution. These modifications jointly improve training convergence.

To evaluate model performance, we used the concordance index (CI) and the integrated Brier score (IBS). CI measures the consistency between the model’s risk scores and the actual occurrence times of events, with higher CI values indicating better model discrimination. IBS assesses predictive accuracy by calculating the mean squared difference between the empirical cumulative event-free curve and the predicted survival curves for each patient. We used the EvalSurv method from the PyCox library version 0.2.3 to compute both CI and IBS.

### Ethical Considerations

The study protocol was reviewed and approved by the institutional review board of Yantai Yuhuangding Hospital (file no 2024-018). All the research data have been deidentified.

## Results

### Characteristics of Study Participants

This study included 189,462 eligible patients from both the United States and China, with 70,370 cases from MIMIC-IV (US cohort; Figure 1A), 112,457 cases from eICU-CRD (US cohort; Figure 1B), 4635 cases from YHD-HOSP (China cohort; Figure 1C), and 1982 cases from SCZG-HOSP (China cohort; Figure 1D). The sample selection process is detailed in Text S1 in Multimedia Appendix 1. Demographic and clinical characteristics of the patients are presented in Tables 1-6. Baseline characteristics stratified by serum chloride levels are presented in Tables S2-5 in Multimedia Appendix 1, demonstrating the baseline features and in-hospital mortality of patients with different serum chloride levels across 4 analytical databases. These findings validate the association between serum chloride levels and mortality risk in critically ill patients, elucidating the population distribution characteristics and prognostic implications and thus providing a basis for identifying high-risk populations and developing clinical intervention strategies.

**Table 1:** Baseline characteristics of the study population.

Variable			MIMIC-IV^a^ (N=70,370)		eICU-CRD^b^ (N=112,457)		YHD-HOSP^c^ (N=4653)		SCZG-HOSP^d^ (N=1982)
In-hospital mortality, n (%)			7677 (10.9)		9805 (8.7)		1445 (31.1)		117 (5.9)
**Sex,** **n (%)**									
	Male	39,417 (56.0)		60,835 (54.1)		2879 (61.9)		1199 (60.5)	
**Race or ethnicity,** **n (%)**									
	White	45,095 (65.1)		86,394 (76.8)		2 (0.0)		—^e^	
	Black	7468 (10.6)		12,418 (11.1)		—		—	
	Other	17,807 (25.3)		13,645 (12.1)		4651 (99.9)		1982 (100)	
**Marital status,** **n (%)**									
	Unknown	7381 (10.5)		—		—		—	
	Divorced	5041 (7.2)		—		12 (0.3)		—	
	Married	30,090 (42.8)		—		4390 (94.3)		—	
	Single	19,434 (27.6)		—		107 (2.3)		—	
	Widowed	8424 (12.0)		—		144 (3.1)		—	
**Smoke,** **n (%)**									
	Yes	5095 (7.2)		296 (0.3)		1320 (28.4)		—	
Age (years), median (IQR)			66.9 (55.1-77.9)		64.00 (53.0-75.0)		66.0 (56.00-74.0)		69.0 (56.0-78.0)
SOFA^f^ scores, median (IQR)			1.0 (0-3)		—		4.0 (2.0-7.0)		—
APACHE IV^g^ scores, median (IQR)			—		52.0 (38.0-69.0)		—		—
Length of stay (days), median (IQR)			6.0 (3.8-10.6)		4.9 (2.8-8.6)		10.0 (6.0-17.0)		9.0 (2.0-21.0)

^a^MIMIC-IV: Medical Information Mart for Intensive Care IV.

^b^eICU-CRD: eICU Collaborative Research Database.

^c^YHD-HOSP: Yantai Yuhuangding Hospital affiliated with Qingdao University.

^d^SCZG-HOSP: Sichuan Zigong Fourth People’s Hospital.

^e^Not available.

^f^SOFA: Sequential Organ Failure Assessment.

^g^APACHE IV: Acute Physiology and Chronic Health Evaluation IV.

**Table 2:** Underlying diseases of the study population.

Variable	MIMIC-IV^a^ (N=70,370)	eICU-CRD^b^ (N=112,457)	YHD-HOSP^c^ (N=4653)	SCZG-HOSP^d^ (N=1982)
Obesity, n (%)	3749 (5.3)	848 (0.8%)	—^e^	—
CHD^f^, n (%)	10,588 (15.1)	5330 (4.7)	180 (3.9)	196 (9.9)
Hypertension, n (%)	13,070 (18.6)	14,554 (12.9)	2432 (52.3)	653 (32.9)
Diabetes, n (%)	9073 (12.9)	11,685 (10.4)	1259 (27.1)	300 (15.1)
Hyperlipidemia, n (%)	13,074 (18.6)	3151 (2.8)	102 (2.2)	82 (4.1)
COPD^g^, n (%)	4133 (5.9)	7604 (6.8)	20 (0.4)	239 (12.1)
Cirrhosis, n (%)	3168 (4.5)	1026 (0.9)	72 (1.5)	—
Hepatic failure, n (%)	1586 (2.3)	291 (0.3)	21 (0.5)	239 (12.1)
Fatty liver, n (%)	370 (0.5)	—	450 (9.7)	152 (7.7)
ICH^h^, n (%)	1669 (2.4)	375 (0.3)	267 (5.7)	309 (15.6)
Cerebral infarction, n (%)	1770 (2.5)	6118 (5.4)	768 (16.5)	184 (9.3)
Malignancy, n (%)	4382 (6.2)	4629 (4.1)	520 (11.2)	—
Solid tumors, n (%)	18,552 (26.4)	—	576 (12.4)	—

^a^MIMIC-IV: Medical Information Mart for Intensive Care IV.

^b^eICU-CRD: eICU Collaborative Research Database.

^c^YHD-HOSP: Yantai Yuhuangding Hospital affiliated with Qingdao University.

^d^SCZG-HOSP: Sichuan Zigong Fourth People’s Hospital.

^e^Not available.

^f^CHD: coronary heart disease.

^g^COPD: chronic obstructive pulmonary disease.

^h^ICH: intracerebral hemorrhage disease.

**Table 3:** Intensive care unit complications of the study population.

Variable	MIMIC-IV^a^ (N=70,370)	eICU-CRD^b^ (N=112,457)	YHD-HOSP^c^ (N=4653)	SCZG-HOSP^d^ (N=1982)
Sepsis, n (%)	32,927 (46.8)	13,111 (11.7)	263 (5.5)	407 (20.5)
ARDS^e^, n (%)	250 (0.4)	830 (0.74)	57 (1.2)	80 (4.0)
Kidney failure, n (%)	6524 (9.3)	10,145 (9.0)	414 (8.9)	418 (21.1)
Acute pancreatitis, n (%)	422 (0.6)	288 (0.3)	25 (0.5)	38 (1.9)
Respiratory failure, n (%)	9509 (13.5)	364 (0.3)	774 (16.6)	788 (39.8)

^a^MIMIC-IV: Medical Information Mart for Intensive Care IV.

^b^eICU-CRD: eICU Collaborative Research Database.

^c^YHD-HOSP: Yantai Yuhuangding Hospital affiliated with Qingdao University.

^d^SCZG-HOSP: Sichuan Zigong Fourth People’s Hospital.

^e^ARDS: acute respiratory distress syndrome.

**Table 4:** Electrolyte levels of the study population.

Variable	MIMIC-IV^a^ (N=70,370), median (IQR)	eICU-CRD^b^ (N=112,457), median (IQR)	YHD-HOSP^c^ (N=4653), median (IQR)	SCZG-HOSP^d^ (N=1982), median (IQR)
Bicarbonate (mEq/L)	24.0 (22-27)	24.0 (22-27)	21.2 (19.2-23.2)	21.4 (18.7-24.0)
Calcium (mEq/L)	8.4 (7.9-8.8)	8.2 (7.8-8.7)	8.5 (8-9)	8.6 (8.0-9.1)
Chloride (mEq/L)	104.0 (100-108)	105.0 (100-108)	105.8 (102.4-108.7)	102.8 (98.8-106.1)
Potassium (mEq/L)	4.1 (3.7-4.5)	4.0 (3.7-4.4)	4.1 (3.7-4.4)	3.7 (3.2-4.2)
Phosphate (mg/dL)	3.5 (2.9-4.2)	NA^e^	3.4 (2.7-3.9)	3.1 (2.4-4.2)
Magnesium (mg/dL)	2.0 (1.7-2.2)	NA	2.1 (1.9-2.3)	2.0 (1.8-2.2)

^a^MIMIC-IV: Medical Information Mart for Intensive Care IV.

^b^eICU-CRD: eICU Collaborative Research Database.

^c^YHD-HOSP: Yantai Yuhuangding Hospital affiliated with Qingdao University.

^d^SCZG-HOSP: Sichuan Zigong Fourth People’s Hospital.

^e^Not available.

**Table 5:** Biochemical parameters of the study population.

Variable	MIMIC-IV^a^ (N=70,370), median (IQR)	eICU-CRD^b^ (N=112,457), median (IQR)	YHD-HOSP^c^ (N=4653), median IQR)	SCZG-HOSP^d^ (N=1982), median (IQR)
Urea nitrogen (mg/dL)	19.0 (13.0-31.0)	19.0 (13.0-31.0)	19.6 (13.2-36.6)	19.1 (13.8-29.8)
Creatinine (mg/dL)	0.9 (0.7-1.4)	1.0 (0.7-1.5)	0.9 (0.7-1.3)	0.9 (0.6-1.5)
WBC^e^ (10^9^/L)	9.7 (7.0-12.2)	10.7 (7.6-15.2)	10.7 (7.8-14.1)	66.8 (45.3-112.5)
Hemoglobin (g/dL)	10.44 (9.0-11.7)	11.1 (9.4-12.6)	11.7 (9.9-13.5)	—^f^
Platelet (10^9^/L)	210.0 (149-264)	197.0 (147-249)	179.0 (130-223)	—
INR^g^	1.3 (1.1-1.5)	—	1.1 (1.1-1.2)	1.2 (1.1-1.4)
Glucose (mg/dL)	128.0 (105-166)	127.00 (103-163)	151.4 (120.7-192.8)	162.2 (126.1-216.2)

^a^MIMIC-IV: Medical Information Mart for Intensive Care IV.

^b^eICU-CRD: eICU Collaborative Research Database.

^c^YHD-HOSP: Yantai Yuhuangding Hospital affiliated with Qingdao University.

^d^SCZG-HOSP: Sichuan Zigong Fourth People’s Hospital.

^e^WBC: white blood cell.

^f^Not available.

^g^INR: international normalized ratio.

**Table 6:** Vital signs of the study population.

Variable	MIMIC-IV^a^ (N=70,370), median (IQR)	eICU-CRD^b^ (N=112,457), median (IQR)	YHD-HOSP^c^ (N=4653), median (IQR)	SCZG-HOSP^d^ (N=1982), median (IQR)
Heart rate (beats/min)	86.0 (75.0-101.0)	88.0 (74.0-100.0)	89.0 (75.0-99.0)	101.0 (82.0-117.0)
DBP^e^ (mm Hg)	67.0 (57.0-79.0)	68.0 (57.0-78.0)	74.0 (63.0-85.0)	77.0 (63.0-89.0)
MBP^f^ (mm Hg)	82.0 (72.0-95.0)	85.0 (72.0-94.0)	94.0 (82.0-105.0)	96.0 (80.0-110.0)
S_P_O_2_^g^ (%)	98.0 (95.0-100.0)	97.0 (96.0-99.0)	97.0 (95.0-99.0)	97.0 (94.0-99.0)
Resp rate (breaths/min)	18.0 (15.0-22.0)	16.0 (20.0-22.0)	18.0 (15.0-21.0)	20.00 (16.0-24.0)
Temperature (°C)	36.7 (36.4-37.1)	36.7 (36.4-37.1)	36.4 (36.0-36.8)	—^h^

^a^MIMIC-IV: Medical Information Mart for Intensive Care IV.

^b^eICU-CRD: eICU Collaborative Research Database.

^c^YHD-HOSP: Yantai Yuhuangding Hospital affiliated with Qingdao University.

^d^SCZG-HOSP: Sichuan Zigong Fourth People’s Hospital.

^e^DBP: diastolic blood pressure.

^f^MBP: mean blood pressure.

^g^SpO_2_: peripheral oxygen saturation.

^h^Not available.

The median (IQR) serum chloride concentration at admission for ICU patients was 104 (100-108) mEq/L for the MIMIC-IV, 105 (100-108) mEq/L for the eICU-CRD, 105.8 (102.4-108.7) mEq/L for the YHD-HOSP, and 102.8 (98.8-106.1) mEq/L for the SCZG-HOSP. Among 70,370 critically ill patients in the MIMIC-IV cohort, 62,693 (89.1%) survived during hospitalization. The survival rates for the eICU-CRD, YHD-HOSP, and SCZG-HOSP cohorts were 91.3%, 68.9%, and 94.1%, respectively.

### Causal Discovery Identifies Mortality-Associated Variables

We conducted causal graph analysis using MIMIC-IV data to identify potential causal relationships between clinical variables collected at ICU admission and patient survival during hospitalization. The analysis revealed a significant causal relationship between serum chloride levels and mortality outcomes (Figure 2). Figure 2A presents demographic variables (race), Figure 2B shows underlying diseases, Figure 2C displays critical diseases, Figure 2D illustrates ions and electrolytes in blood, Figure 2E details blood biochemical indicators, and Figure 2F summarizes vital signs. In addition, we identified 32 other clinical variables that have potential causal associations with the in-hospital mortality, such as age, SOFA score, the presence of sepsis, liver failure, and serum calcium ions (Figure 2).

### Cox Regression Confirms Mortality Risk

**Figure 4:**
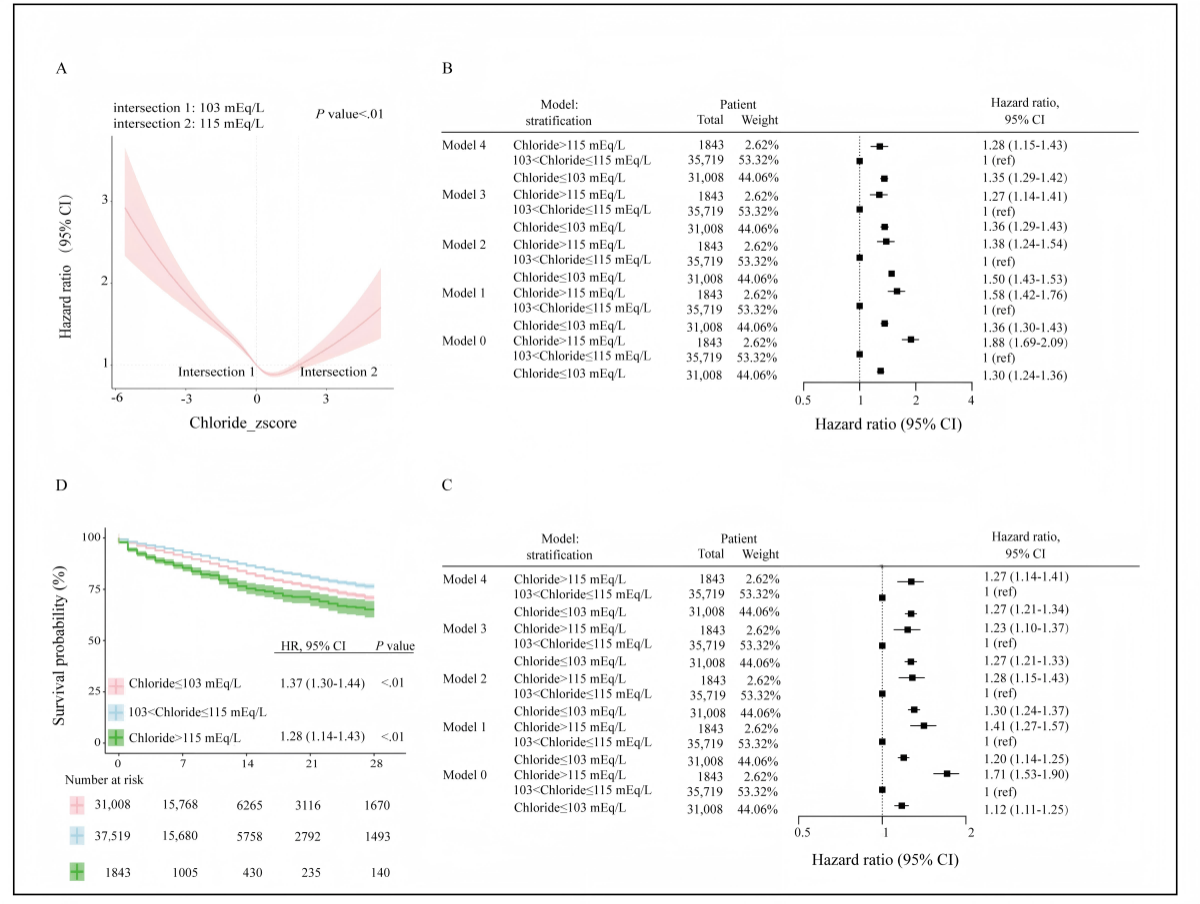
Association between serum chloride levels at intensive care unit admission and in-hospital mortality based on Medical Information Mart for Intensive Care IV (MIMIC-IV) data: (A) restricted cubic spline analysis reveals a U-shaped association between serum chloride concentration and in-hospital mortality, with the thresholds of 103 and 115 mEq/L (*P*<.01); (B) Cox regression analysis demonstrates the hazard ratios for in-hospital mortality across serum chloride ranges, with 103-115 mEq/L as the reference category; (C) Cox frailty model results, adjusting for intensive care unit type, confirm the association between extreme chloride levels and increased mortality risk; and (D) Kaplan-Meier survival curves illustrate significantly lower 28-day survival probabilities for patients with chloride levels ≤103 mEq/L or >115 mEq/L compared with those in the 103-115 mEq/L range. HR: hazard ratio.

The RCS analysis revealed a U-shaped association between serum chloride levels and HRs in ICU patients (*P*<.01; Figure 4A), with critical thresholds at 103 and 115 mEq/L, a finding corroborated in the YHD-HOSP cohort (Figure S3 in Multimedia Appendix 1).

To further explore the relationship between admission serum chloride concentration and in-hospital mortality, we stratified MIMIC-IV patients based on the chloride thresholds identified in the RCS analysis. This cohort included 37,519 ICU patients with serum chloride levels ranging between 103 and 115 mEq/L at admission; 31,008 patients had chloride levels ≤103 mEq/L, while only 1843 patients had chloride levels >115 mEq/L.

We constructed 1 unadjusted and 4 adjusted Cox regression and Cox frailty models, considering confounding factors and ICU heterogeneity. Compared to the reference range of 103-115 mEq/L, patients with chloride levels above the upper limit (>115 mEq/L) or below the lower limit (≤103 mEq/L) exhibited a significantly increased risk of in-hospital mortality in both unadjusted and adjusted models (Figure 4B). After adjusting for ICU heterogeneity, the increased mortality risk in the Cox frailty model remained consistent, indicating robust results despite differences between ICUs (Figure 4C). In addition, Kaplan-Meier survival curves were generated to visualize survival probabilities among patients with different chloride levels. Overall, patients with chloride levels ≤103 mEq/L or >115 mEq/L had lower survival probabilities compared to those with chloride levels between 103 and 115 mEq/L (Figure 4D).

### Cross-Cohort and Cross-Country Validation

Furthermore, our findings were validated using datasets from eICU-CRD, YHD-HOSP, and SCZG-HOSP. Patients in these cohorts were stratified based on the chloride thresholds related to mortality identified in the MIMIC-IV cohort. In eICU-CRD, the number of ICU patients with serum chloride levels ≤103 mEq/L, 103-115 mEq/L, and >115 mEq/L was 47,365, 61,605, and 3487, respectively. In the YHD-HOSP cohort, 3090 patients had chloride levels between 103-115 mEq/L at ICU admission, 1375 patients had chloride levels ≤103 mEq/L, and 118 patients had chloride levels >115 mEq/L. In the SCZG-HOSP cohort, 911 patients had chloride levels between 103 and 115 mEq/L at ICU admission, 1024 patients had chloride levels ≤103 mEq/L, and only 47 patients had chloride levels >115 mEq/L.

Consistent with the MIMIC-IV results, compared to the reference range (103-115 mEq/L), patients with serum chloride levels ≤103 mEq/L had an adjusted HR of 1.30 (95% CI 1.24-1.36) (Figure S4A in Multimedia Appendix 1). Similarly, in the YHD-HOSP cohort, using the 103-115 mEq/L chloride range as a reference, patients with chloride levels ≤103 mEq/L had an HR of 1.23 (95% CI 1.09-1.38) in adjusted models, while those with levels >115 mEq/L had a risk of 1.58 (95% CI 1.27-1.96) (Figure S4C in Multimedia Appendix 1). In the SCZG-HOSP database, the in-hospital mortality risk for patients with chloride levels ≤103 mEq/L was 2.20 (95% CI 1.43-3.39) (Figure S4E in Multimedia Appendix 1). In addition, Kaplan-Meier survival curves show survival differences between chloride of the eICU-CRD, YHD-HOSP, and SCZG-HOSP cohorts (Figures S4B, 4D, and 4F in Multimedia Appendix 1), mirroring the findings of the MIMIC-IV cohort. Multinational validation demonstrates significant associations between serum chloride thresholds (≤103 and >115 mEq/L) and ICU mortality, establishing their independent prognostic value for risk stratification and clinical decision-making.

### Sensitivity Analyses Confirm Findings’ Robustness

To further validate the robustness of our model, we evaluated the impact of AKI on the association between serum chloride levels and in-hospital mortality by incorporating AKI into the model for sensitivity analysis. Consistent with the primary estimates, after additional adjustment for AKI in the Cox regression model, the increased mortality risk associated with chloride levels below the lower limit or above the upper limit, compared to the 103-115 mEq/L range, remained significant (Figure S5 in Multimedia Appendix 1). These findings reinforce chloride’s reliability as an independent prognostic marker, suggesting clinicians should monitor chloride abnormalities for risk stratification and treatment optimization, even in patients with AKI.

In addition to AKI, we further incorporated sodium as another sensitivity analysis indicator. Consistent with prior findings, even after adjusting for sodium levels in the Cox regression model, the 2 subgroups with out-of-threshold chloride levels (103-115 mEq/L) continued to exhibit significantly elevated mortality risks (Figure S6 in Multimedia Appendix 1), corroborating prior studies that serum chloride independently predicts outcomes and supporting its routine monitoring in clinical management.

### Modeling Personalized ICU Survival Predictions

Using the MIMIC-IV dataset, we developed the Causal SurvivalNet, a deep learning model based on ICU admission serum chloride levels and mortality-related variables to generate personalized 28-day hospital survival curves. Our model adapts the traditional CPHs framework by replacing prespecified coefficients with learnable multilayer perceptron weights. Additional nonlinear ReLU activation and batch normalization layers enable robust fitting to the intensive care data (Figure 3A).

To facilitate clinical use, we converted the Causal SurvivalNet to a web tool (Figure 3B). The webpage layout is simple and easy to use. Users can upload patient data by clicking the input button in the top-left corner, then clicking the “Run” button in the top-right corner to instantly generate the patient’s survival curve for real-time risk prediction. The data can then be saved using the output function (Figure 3B). We show the representative curves for patients stratified by three defined chloride ranges in Figures 3C-3E. The IBS, ranging from 0 (best) to 1 (worst), was used to evaluate model performance. For chloride levels ≤103 mEq/L, 103-115 mEq/L, and >115 mEq/L, the IBS values for our personalized survival curve model, Causal SurvivalNet, were 0.09, 0.12, and 0.15, respectively, reflecting strong overall predictive capability.

## Discussion

### Principal Findings

Critically ill patients often experience chloride imbalance, disrupting physiology and increasing complications. In this large-scale, cross-country study involving four major datasets from the United States and China, we identified a causal relationship between serum chloride concentration and in-hospital mortality in critically ill patients. Using rigorous statistical and computational methods, we established a novel prognostic serum chloride range of 103-115 mEq/L associated with mortality outcomes in ICU patients. Further Cox regression and fragility models revealed significantly increased risk of in-hospital death among patients with admission chloride measurements ≤103 mEq/L or >115 mEq/L compared to the 103-115 mEq/L reference range. This U-shaped relationship and the identified thresholds were validated across all four cohorts and in both countries. Furthermore, we developed Causal SurvivalNet, an innovative deep learning model that generates individualized survival curves during ICU stay, incorporating the chloride thresholds and other causal factors. Overall, our data-driven approach provides robust, clinically actionable chloride thresholds (103-115 mEq/L range) and AI models, which enable earlier risk assessment and intervention for ICU patients.

Chloride imbalance can disrupt physiological processes such as electrolyte homeostasis and cell signaling [29,30], which may lead to complications including seizures [31], arterial hypertension [32], acute kidney failure [33,34], and heart failure [35,36]. While prior studies have examined the associations between clinical factors and outcomes in critically ill patients [37-39], they have been limited by small sample sizes in single centers, which lack model interpretability. Using causal analysis models, we discovered a substantive causal relationship between serum chloride levels and mortality probability in ICU patients. Our approach enables more intuitive insights into these exposure-outcome relationships compared to traditional methods. Overall, our data-driven causal methodology provides novel evidence linking chloride imbalance to survival outcomes across diverse patient cohorts and care settings. The findings suggest chloride could be a modifiable risk factor, and the causal modeling techniques can be extended to other prognostic factors in critical care research.

Compared to healthy individuals, ICU patients have diminished physiological reserves, making them vulnerable to complications from chloride fluctuations that can increase hospital stay and mortality [6,30,40]. Rather than relying on the physiologically normal chloride range in adults (96-108 mEq/L) for prognosis assessment [4-6], we leveraged robust statistical methods to establish a new prognostic serum chloride interval of 103-115 mEq/L associated with in-hospital mortality across four large cohorts in two countries. Interestingly, the serum chloride thresholds associated with increased mortality risk differ from common physiological reference ranges.

Compared to normal chloride in healthy adults, this data-driven range may allow more accurate, early risk assessment for critically ill patients. For example, ICU patients with admission serum chloride between 96 and 103 have an increased risk of 28-day in-hospital mortality, even though their chloride levels may still be within the physiologically “normal” range. Similarly, levels between 108 and 115 may still have better survival probabilities that warrant resource allocation, despite exceeding the upper limit of the physiological normal threshold. Moreover, unlike previous assumptions [37], we revealed a U-shaped, nonlinear association between chloride levels and mortality. While traditional survival analysis captures population-level trends, its ability to predict individual patient outcomes remains limited [41]. In this study, we developed a personalized survival analysis model to generate individual survival curves for ICU patients. Our deep learning model incorporates batch normalization and nonlinear ReLU activation functions to address overfitting and vanishing gradients [42]. We also introduced nonlinearity within the network to improve convergence. We implemented the optimized Causal SurvivalNet model on the web. This advancement provides more precise, individual-level prognostic insights to guide therapy adjustments for ICU patients. The accessible web implementation significantly enhances clinical utility and supports future research.

### Limitations

However, some limitations for this study should be noted. First, the Chinese cohort’s small sample size, short data collection period, and missing data necessitate future large-scale longitudinal studies. In addition, variations in severity scoring systems (SOFA/APACHE IV) and missing scores in one dataset may introduce bias, though we mitigated this through covariate adjustments. The consistent findings across cohort’s support robustness, but future research should standardize scoring or use advanced methods to further address potential biases. Second, as an observational study, despite its comprehensive design and the robustness of results verified by sensitivity analysis, unmeasured confounding factors (such as genetic predispositions to chloride metabolism, unrecorded comorbidities, inconsistent records of chloride-containing fluids or medications, differences in nursing quality across ICUs and hospitals, and undiagnosed acute diseases at ICU admission) may affect the accuracy of results. Although the chloride threshold (103-115 mEq/L) and associated mortality risk determined in this study are reliable, they may not fully reflect the complex relationships in all clinical settings. Future research should incorporate data on potential confounding factors to further clarify the causal relationships in critically ill patients. Third, the study used probabilistic principal component analysis to address missing data in continuous variables with less than 20% missingness, though explicit sensitivity analyses on imputation were not conducted. Nevertheless, cross-cohort validation across multiple international datasets inherently provided a robustness check; future work needs to incorporate more detailed sensitivity assessments to further enhance the analysis. Finally, this study only focuses on the static chloride levels of critically ill patients at ICU admission, without considering their dynamic changes. Future research should focus on the fluctuations of chloride levels over time and their impact on patient prognosis.

### Conclusion

In conclusion, this large-scale study of 189,462 patients from 4 cohorts across 2 countries uses robust statistical analysis, causal inference, and innovative deep learning to establish a U-shaped relationship between serum chloride (103-115 mEq/L) and in-hospital mortality risk in patients who are critically ill, validated across datasets. We also developed Causal SurvivalNet, a personalized deep learning model generating individualized survival curves to enhance precision prognosis in ICU care. Overall, this study establishes a significant foundation for improving outcomes through data-driven, personalized medicine in critical care. Future research should externally validate these findings and integrate emerging clinical variables such as omics data to further refine prognosis.

## Appendix

Multimedia Appendix 1 Supplementary material.

## Abbreviations

AKI: acute kidney injury
APACHE IV: Acute Physiology and Chronic Health Evaluation IV
CI: concordance index
CPH: Cox proportional hazards
eICU-CRD: eICU Collaborative Research Database
FCI: fast causal inference
HR: hazard ratio
IBS: integrated Brier score
ICD-10: International Classification of Diseases and Related Health Problems, Tenth Revision
ICU: intensive care unit
MIMIC-IV: Medical Information Mart for Intensive Care IV
RCS: restricted cubic spline
ReLU: rectified linear unit
SCZG-HOSP: Sichuan Zigong Fourth People’s Hospital
SOFA: Sequential Organ Failure Assessment
WHO: World Health Organization
YHD-HOSP: Yantai Yuhuangding Hospital affiliated with Qingdao University

## Data Availability

The data sets used and analyzed during this study are available from the corresponding author upon reasonable request.
