# Supplementary material for: Predicting In-Hospital Mortality in Intensive Care Unit Patients Using Causal SurvivalNet With Serum Chloride and Other Causal Factors: Cross-Country Study
Source: J Med Internet Res. 2025 Jul 24;27:e70118. doi: 10.2196/70118 (PMC12332460; doi:10.2196/70118)
Supplement: Multimedia Appendix 1 [file jmir_v27i1e70118_app1.docx]

| **Text S1** | Detailed information of participant selection | Page 2 |
| --- | --- | --- |
| **Text S2** | The description of FCI | Page 4 |
| **Text S3** | The mathematical principles of CPH. | Page 5 |
| **Text S4** | The Cox frailty model | Page 5 |
| **Text S5** | The traditional CPH model | Page 5 |
| **Text S6** | The neural network | Page 5 |
| **Text S7** | The learning process of the deep survival analysis | Page 6 |
| **Table S1** | The variables included in the MIMIC-IV cohort | Page 8 |
| **Table S2** | **Stratify baseline characteristics by serum chloride levels in the MIMIC-IV database** | Page 9 |
| **Table S3** | **Stratify baseline characteristics by serum chloride levels in the eICU-CRD database** | Page 9 |
| **Table S4** | **Stratify baseline characteristics by serum chloride levels in the YHD-HOSP database** | Page 10 |
| **Table S5** | **Stratify baseline characteristics by serum chloride levels in the SCZG-HOSP database** | Page 11 |
| **Figure S1** | Analysis flow chart | Page 13 |
| **Figure S2** | RCS curve for sodium in the MIMIC cohort | Page 16 |
| **Figure S3** | Based on RCS diagram in YHD-HOSP database | Page 17 |
| **Figure S4** | Cross-cohort and cross-country validation of serum chloride thresholds  associated with in-hospital mortality | Page 18 |
| **Figure S5** | Sensitivity analyses confirming the robustness of the association between serum chloride levels and in-hospital mortality after adjusting for Acute Kidney Injury (AKI) | Page 20 |
| **Figure S6** | Sensitivity analyses validating the consistency of the relationship between serum chloride concentrations and in-hospital mortality after sodium adjustment | Page 21 |

**Supplementary Material Online**

**Text S1.** **Detailed information of participant selection.**

Definition of inclusion criteria

- Study Population: Patients admitted to the ICU.
- Blood Test Indicators: All blood test data are based on the initial measurements taken upon ICU admission.
- Observation Period: The observation period is defined as the time from ICU admission to patient discharge.
- Outcome Alignment: In-hospital mortality and survival status are aligned with the patient’s discharge status.
- Missing Data Handling: Continuous variables with more than 20% missing data are excluded from the analysis.

The workflow of sample selection

1. MIMIC-IV Database (Version 2.2)
   Total records: 431,213
   - Excluded 360,792 non-ICU records
   - Included 70,445 (16.3%) ICU patients
   - Further exclusions: 66 incorrect chloride records, 9 patients with negative ICU entry time,

Final MIMIC-IV cohort: 70,370 patients

1. Philips eICU-CRD Database

Total records: 200,859

- - Excluded:
    - 50,767 (25.4%) patients with missing chloride data
    - 1,579 (0.8%) patients with incorrect or missing leaving ICU status
    - 36,056 (18.0%) patients with missing or negative APACHE IV scores

Final eICU-CRD cohort: 112,457 patients

1. Yuhuangding Hospital Database (YHD-HOSP)

Total records: 11,095

- - Excluded:
    - 2,784 (25.1%) patients with missing chloride data
    - 3,658 (33.0%) patients with missing SOFA

Final YHD-HOSP cohort: 4,653 patients

1. Zigong Fourth People’s Hospital (SCZG-HOSP)

Total records: 2,790

- - Excluded:
    - 808 (29.0%) patients with missing chloride data

Final SCZG-HOSP cohort: 1,982 patients

Total study population across all three databases: 189,462 patients

Database Descriptions:

1. MIMIC-IV: Established in 2003 by the Computational Physiology Laboratory at MIT, in collaboration with Beth Israel Deaconess Medical Center (BIDMC) at Harvard Medical School and Philips Medical, MIMIC-IV provides a comprehensive dataset of ICU patient information.
2. eICU-CRD: This database includes data from multiple ICUs across 20 large U.S. hospitals, collected between 2014 and 2015. It encompasses a variety of ICU types, including cardiology, thoracic, internal medicine, and neurosurgery, offering a diverse patient population for research.
3. YHD-HOSP: Affiliated with Qingdao University in China, YHD-HOSP is a tertiary grade A hospital. Its intensive care department contributed clinical data for validation against the US databases.
4. SCZG-HOSP：This dataset was collected from the Fourth People’s Hospital of Zigong between December 2016 and June 2019 to facilitate cross-verification with the US database.

**Text S2. The description of FCI**

The Fast Causal Inference (FCI) algorithm is a robust tool for inferring causal relationships from observational data, especially in the presence of latent confounders. Two-stage adjacency-orientation process.

1. Adjacency Stage:

In this initial phase, the FCI algorithm examines pairs of variables to identify potential causal connections. It performs conditional independence tests on subsets of observed variables to eliminate edges between adjacent variables that are conditionally independent. Crucially, the algorithm records the conditioning sets that lead to the removal of these edges, as this information is vital for the subsequent orientation stage.

2. Orientation Stage:

Utilizing the stored conditioning sets from the adjacency stage, the algorithm then seeks to orient as many edges as possible. This process involves applying a series of orientation rules to establish the directionality of causal relationships. The FCI algorithm is capable of distinguishing between direct causal relationships, indirect relationships, and potential latent confounders.

Key Features of FCI.

1. Handles latent confounders: Unlike some other causal discovery algorithms, FCI can infer causal relationships even in the presence of unmeasured common causes.

2. Produces a Partial Ancestral Graph (PAG): The output of FCI is a PAG, which represents the class of causal models consistent with the observed data.

3. Soundness and completeness: Under certain assumptions, FCI is both sound (all inferred causal relationships are correct) and complete (all identifiable causal relationships are inferred).

Application in This Study:

To enhance the efficiency and accuracy of our causal analysis, we applied the FCI algorithm to six categories of variables:

1. Demographic data

2. Critical illness indicators

3. Potential underlying diseases

4. Blood ions and electrolytes

5. Biochemical indicators

6. Vital signs

**Text S3. The Mathematical Principles of CPH**

$$h\left( t | x \right)=h_{0}\left( t \right)\cdot e^{\beta_{1}x_{i1}+\beta_{2}x_{i2}+\cdots+\beta_{p}x_{ip}}$$

Where, h(t|x) represents the risk of an event occurring at time t given the independent variables (i.e., the hazard function).

h_0 (t) is the baseline hazard function, which represents the risk of an event occurring at time t when all the independent variables are 0.

β_1_, β_2_, ⋯ , β_p_ are the regression coefficients corresponding to the independent variables, which quantify the impact of the independent variables on the hazard function.

x_i1_, x_i2_, ⋯ , $x_{ip}$ are the independent variables.

**Text S4. The Cox frailty model**

$h_{ij}\left( t | X \right)=\mu_{i}h_{0}\left( t | X \right)\exp\left( X\beta\right)$ $(1)$

Where $\mu_{i}$ represents the frailty term for each ICU, assumed to follow a distribution with mean 1 and independent of $X$. When $\mu_{i}=1$, the model simplifies to Cox regression.

**Text S5. The traditional CPH model**

$$h\left( t | x \right)=h_{0}(t)\cdot e^{\beta^{T}X^{T}} (2)$$

Where $\beta^{T}$ and $X^{T}$ represent coefficient vectors and chloride value vectors, respectively.

**Text S6. The neural network**

$$z^{l}=\theta_{1}^{l}x_{1}^{l-1}+\theta_{2}^{l}x_{2}^{l-1}+\cdots+\theta_{n}^{l}x_{n}^{l-1}+b (3)$$

In this equation, each product term represents the fundamental unit of the CPH model ($\beta^{T}X^{T}$). $z^{l}$ denotes the output of the l-th layer, and $\theta_{n}^{l}x_{n}^{l-1}$ represents the weighted sum of $x_{n}^{l-1}$ from the (l-1)-th layer using the weights $\theta_{n}^{l}$, where $n\in(1,2,3,\cdots,n)$. Here, $x$ is the known variable (covariate/exposure factor), the network weights $\theta$ are the unknowns, $z$ represents the output, and $b$ is the bias.

To enhance the model’s nonlinearity, we use$h\left( t | x \right)=h_{0}(t)\cdot e^{a(\theta,x)}$, where $a(\theta,x)$ represents the activation function of the neural network.

**Text S7. The learning process of the deep survival analysis**

$$d_{\theta^{l}}=\frac{\partial z}{\partial_{\theta^{l}}}+\frac{\lambda}{m}\theta^{l} (4)$$

$$\hat{\theta}^{l}=\theta^{l}-\alpha d_{\theta^{l}}=\theta^{l}-\alpha\frac{\partial z}{\partial_{\theta^{l}}}-\alpha\frac{\lambda}{m}\theta^{l}=\theta^{l}-\alpha\frac{\lambda}{m}\theta^{l}-\alpha\frac{\partial z}{\partial_{\theta^{l}}} (5)$$

$$\hat{\theta}^{l}=\left( 1-\alpha\frac{\lambda}{m} \right)\theta^{l}-\alpha\frac{\partial z}{\partial_{\theta^{l}}} (6)$$

Where $\alpha$ is the learning rate. $\frac{\lambda}{m}$ is the constant obtained by taking the partial derivative of L2 regularization.

To accelerate model convergence, we added a rectified linear unit (ReLU) activation function and a batch normalization layer after the FC layer. The ReLU activation function is defined as$y=max(0, x)$, where $x$ is the input value and $y$ is the output. The ReLU function simplifies computation, facilitates gradient calculation, and helps alleviate the vanishing gradient problem. Additionally, the batch normalization layer normalizes input data within each batch to a standard normal distribution with mean 0 and variance 1, mitigating the vanishing gradient issue.

**Table S1. The variables included in the MIMIC-IV cohort**

| **Demographic variables** | **Critical illness** | **Underlying diseases** | **Ions and electrolytes** | **Biochemical indicators** | **Vital signs** | **Critical Care Scoring Systems** |
| --- | --- | --- | --- | --- | --- | --- |
| Sex, Race^a^, Age^a^, Marriage^a^, Smoker | Sepsis^a^, ARDS^a,b^, Kidney failure, Acute pancreatitis, Respiratory failure^a^ | Obesity, CHD^b^, Hypertension^a^, Diabetes, Hyperlipidemia, COPD^a,c^, Cirrhosis, Hepatic failure^a^, Fatty liver, ICH^a,e^, Cerebral infarction^a^, Malignance^a^, Solid tumors^a^ | Calcium^a^, Bicarbonate^a^,  Potassium^a^,  Phosphate^a^,  Magnesium^a^ | BUN^a,f^,  Creatinine^a^, WBC^a,h^, Hemoglobin^a^, Platelet^a^, INR^a,i^, Glucose^a^ | Heart rate^a^, MBP^a,j^,  DBP^a,k^, Respiratory rate^a^, SpO2^a^ | SOFA^a,m^ or APACHE IV^a,n^ |

^a^The variables that were found by the causal graphical inference algorithm were not causally related to 28-day in-hospital mortality.

^b^ARDS: acute respiratory distress syndrome.

^c^CHD: coronary heart disease.

^d^COPD: chronic obstructive pulmonary disease.

^e^ICH: cerebral hemorrhage.

^f^BUN: blood Urea Nitrogen.

^h^WBC: international normalized ratio.

^i^INR: international normalized ratio.

^j^MBP: mean blood pressure.

^k^BDP: diastolic blood pressure.

^l^SpO_2:_ peripheral oxygen saturation.

^m^SOFA: sequential organ failure assessment.

^n^APACHE IV: acute physiology and chronic health evaluation IV.

**Table S2: Stratify baseline characteristics by serum chloride levels in the MIMIC-IV database.**

| Variables | ≤ 103 mEq/L(n = 31,008) | 103 - 115 mEq/L(n =3 7,519) | > 115 mEq/L( n= 1,843) |
| --- | --- | --- | --- |
| In-hospital mortality, n(%) | 4,020 (1.4) | 3277 (4.7) | 380 (0.5) |
| **Sex** |  |  |  |
| Male, n(%) | 17,368 (24.6) | 21,129 (30.0) | 920 (1.3) |
| **Race/ethnicity** |  |  |  |
| White, n(%) | 20,291 (28.8) | 23,735 (33.7) | 1070 (1.5) |
| Black, n(%) | 3713 (5.3) | 3502 (5.0) | 253 (0.4) |
| Other, n(%) | 7004 (10.0) | 10,282 (14.6) | 521 (0.7) |
| **Marital status** |  |  |  |
| Unknown, n(%) | 2355 (3.3) | 4804 (6.8) | 222 (0.3) |
| Divorced, n(%) | 2416 (3.4) | 2522 (3.6) | 103 (0.1) |
| Married, n(%) | 13,131 (18.7) | 16,219 (23.0) | 740 (1.1) |
| Single, n(%) | 9107 (12.9) | 9804 (13.9) | 524 (0.7) |
| Widowed, n(%) | 3999 (5.7) | 4170 (5.9) | 255 (0.4) |
| **Smoke,** n(%) | 2402 (3.4) | 2599 (3.7) | 94 (0.1) |
| **Obesity**, n(%) | 1804 (2.6) | 1858 (2.6) | 87 (0.1) |
| **CHD^a^**, n(%) | 5305 (7.5) | 5120 (7.3) | 163 (0.2) |
| **Hypertension**, n(%) | 6471 (9.2) | 6428 (9.1) | 171 (0.2) |
| **Diabetes**, n(%) | 5174 (7.3) | 3740 (5.3) | 159 (0.2) |
| **Hyperlipidemia**, n(%) | 5991 (8.5) | 6802 (9.7) | 281 (0.4) |
| **COPD^b^**, n(%) | 2590 (3.7) | 1494 (2.1) | 49 (0.1) |
| **Cirrhosis**, n(%) | 1544 (2.2) | 1538 (2.2) | 86 (0.1) |
| **Hepatic failure**, n(%) | 977 (1.4) | 559 (0.8) | 50 (0.1) |
| **Fatty liver**, n(%) | 176 (0.3) | 189 (0.3) | 5 (0.0) |
| **ICH^c^**, n(%) | 845 (1.2) | 787 (1.1) | 37 (0.0) |
| **Cerebral infarction**, n(%) | 855 (1.2) | 855 (1.2) | 30 (0.0) |
| **Malignancy**, n(%) | 2589 (3.7) | 1730 (2.5) | 63 (0.1) |
| **Solid tumors**, n(%) | 9590 (13.6) | 8615 (12.2) | 347 (0.5) |
| **Sepsis**, n(%) | 14138 (20.1) | 17551 (24.9) | 1238 (1.8) |
| **ARDS^d^**, n(%) | 140 (0.2) | 97 (0.1) | 13 (0.0) |
| **Kidney failure**, n(%) | 3250 (4.6) | 3119 (4.4) | 155 (0.2) |
| **Acute pancreatitis**, n(%) | 191 (0.3) | 225 (0.3) | 6 (0.0) |
| **Respiratory failure**, n(%) | 5655 (8.0) | 3665 (5.2) | 189 (0.3) |

^a^CHD: Coronary heart disease.

^b^COPD: Chronic obstructive pulmonary disease.

^c^ICH: Intracerebral hemorrhage disease.

^d^ARDS: Acute respiratory distress syndrome.

**Table S3:** Stratify baseline characteristics by serum chloride levels in the eICU-CRD database.

| Variables | ≤ 103 mEq/L(n = 47,365) | 103 - 115 mEq/L(n =61,605) | > 115 mEq/L( n= 3487) |
| --- | --- | --- | --- |
| In-hospital mortality, n(%) | 4541 (4.0) | 4679 (4.2) | 585 (0.5) |
| **Sex** |  |  |  |
| Male, n(%) | 26,107 (23.2) | 33,027 (29.4) | 1,701 (1.5) |
| **Race/ethnicity** |  |  |  |
| White, n(%) | 36,792 (32.7) | 47,105 (41.9) | 2,497 (2.2) |
| Black, n(%) | 5,484（4.9） | 6,490 (5.8) | 444 (0.4) |
| Other, n(%) | 5,089 (4.5) | 8,010 (7.1) | 546 (0.5) |
| **Smoke** |  |  |  |
| Yes, n(%) | 102 (0.1) | 184 (0.2) | 10 (0.0) |
| **Obesity**, n(%) | 455 (0.4) | 382 (0.3) | 11 (0.0) |
| **CHD^a^**, n(%) | 2200 (2.0) | 3033 (2.7) | 97 (0.1) |
| **Hypertension**, n(%) | 6429 (5.7) | 7825 (7.0) | 300 (0.3) |
| **Diabetes**, n(%) | 5568 (5.0) | 5764 (5.1) | 353 (0.3) |
| **Hyperlipidemia**, n(%) | 1217 (1.1) | 1875 (1.7) | 59 (0.1) |
| **COPD^b^**, n(%) | 4603 (4.1) | 2886 (2.6) | 115 (0.1) |
| **Cirrhosis**, n(%) | 442 (0.4) | 528 (0.5) | 56 (0.0) |
| **Hepatic failure**, n(%) | 160 (0.1) | 120 (0.1) | 11 (0.0) |
| **ICH^c^**, n(%) | 140 (0.1) | 225 (0.2) | 10 (0.0) |
| **Cerebral infarction**, n(%) | 2309 (2.1) | 3652 (3.2) | 157 (0.1) |
| **Malignancy**, n(%) | 2060 (1.8) | 2438 (2.2) | 131 (0.1) |
| **Sepsis**, n(%) | 5155 (4.6) | 7093 (6.3) | 863 (0.8) |
| **ARDS^d^**, n(%) | 335 (0.3) | 454 (0.4) | 41 (0.0) |
| **Kidney failure**, n(%) | 4300 (3.8) | 5133 (4.6) | 712 (0.6) |
| **Acute pancreatitis**, n(%) | 128 (0.1) | 145 (0.1) | 15 (0.0) |
| **Respiratory failure**, n(%) | 234 (0.2) | 120 (0.1) | 10 (0.0) |

^a^CHD: Coronary heart disease.

^b^COPD: Chronic obstructive pulmonary disease.

^c^ICH: Intracerebral hemorrhage disease.

^d^ARDS: Acute respiratory distress syndrome.

**Table S4: Stratify baseline characteristics by serum chloride levels in the YHD-HOSP database.**

| Variables | ≤ 103 mEq/L(n = 1,375) | 103 - 115 mEq/L(n =3,090) | > 115 mEq/L( n= 188) |
| --- | --- | --- | --- |
| In-hospital mortality, n(%) | 558 (12.0) | 784 (16.8) | 103 (2.2) |
| **Sex** |  |  |  |
| Male, n(%) | 893 (19.2) | 1880 (40.4) | 106 (2.3) |
| **Race/ethnicity** |  |  |  |
| White, n(%) | 1 (0.0) | 1 (0.0) | NA |
| Black, n(%) | NA | NA | NA |
| Other, n(%) | 1374 (29.5) | 3089 (66.4) | 188 (4.0) |
| **Marital status** |  |  |  |
| Unknown, n(%) | NA | 2 (0.0) | NA |
| Divorced, n(%) | 4 (0.1) | 13 (0.3) | NA |
| Married, n(%) | 1307 (28.1) | 2915 (62.6) | 179 (3.8) |
| Single, n(%) | 25 (0.5) | 69 (1.5) | 5 (0.1) |
| Widowed, n(%) | 39 (0.8) | 91 (2.0) | 4 (0.1) |
| **Smoke** |  |  |  |
| Yes, n(%) | 437 (9.4) | 991 (21.3) | 62 (1.3) |
| **CHD^a^** n(%) | 57 (1.2) | 116 (2.5) | 7 (0.2) |
| **Hypertension**, n(%) | 734 (15.8) | 1,602 (34.4) | 96 (2.1) |
| **Diabetes** n(%) | 435 (9.3) | 765 (16.4) | 59 (1.3) |
| **Hyperlipidemia**, n(%) | 43 (0.9) | 56 (1.2) | 3 (0.1) |
| **COPD^b^**, n(%) | 17 (0.4) | 3 (0.1) | NA |
| **Cirrhosis**, n(%) | 20 (0.4) | 48 (1.0) | 4 (0.1) |
| **Hepatic failure**, n(%) | 9 (0.2) | 12 (0.3) | NA |
| **Fatty liver**, n(%) | 165 (3.5) | 273 (5.9) | 12 (0.3) |
| **ICH^c^**, n(%) | 64 (1.4) | 172 (3.7) | 31 (0.7) |
| **Cerebral infarction**, n(%) | 215 (4.6) | 523 (11.2) | 30 (0.6) |
| **Malignancy**, n(%) | 104 (2.2) | 401 (8.6) | 15 (0.3) |
| **Solid tumors**, n(%) | 143 (3.1) | 414 (8.9) | 19 (0.4) |
| **Sepsis**, n(%) | 108 (2.3) | 135 (2.9) | 20 (0.4) |
| **ARDS^d^**, n(%) | 14 (0.3) | 40 (0.9) | 3 (0.1) |
| **Kidney failure**, n(%) | 198 (4.3) | 205 (4.4) | 11 (0.2) |
| **Acute pancreatitis**, n(%) | 10 (0.2) | 15 (0.3) | NA |
| **Respiratory failure**, n(%) | 328 (7.0) | 389 (8.4) | 57 (1.2) |

^a^CHD: Coronary heart disease.

^b^COPD: Chronic obstructive pulmonary disease.

^c^ICH: Intracerebral hemorrhage disease.

^d^ARDS: Acute respiratory distress syndrome.

**Table S5: Stratify baseline characteristics by serum chloride levels in the SCZG-HOSP database.**

| Variables | ≤ 103 mEq/L(n =1,024) | 103 - 115 mEq/L(n =911) | > 115 mEq/L( n= 47) |
| --- | --- | --- | --- |
| In-hospital mortality, n(%) | 78 (3.9) | 37 (1.9) | 2 (0.1) |
| **Sex** |  |  |  |
| Male, n(%) | 635 (32.0) | 534 (26.9) | 30 (1.5) |
| **Race/ethnicity** |  |  |  |
| White, n(%) | NA | NA | NA |
| Black, n(%) | NA | NA | NA |
| Other, n(%) | 1,024 (51.7) | 911 (46.0) | 47 (2.4) |
| **CHD^a^**, n(%) | 108 (5.4) | 88 (4.4) | NA |
| **Hypertension**, n(%) | 336 (17.0) | 296 (14.9) | 21 (1.1) |
| **Diabetes**, n(%) | 162 (8.2) | 122 (6.2) | 16 (0.8) |
| **Hyperlipidemia**, n(%) | 43 (2.2) | 37 (1.9) | 2 (0.1) |
| **COPD^b^**, n(%) | 206 (10.4) | 61 (3.1) | 4 (0.2) |
| **Hepatic failure**, n(%) | 126 (6.4) | 111 (5.6) | 2 (0.1) |
| **Fatty liver**, n(%) | 81 (4.1) | 67 (3.4) | 4 (0.2) |
| **ICH^c^**, n(%) | 154 (7.8) | 149 (7.5) | 6 (0.3) |
| **Cerebral infarction**, n(%) | 84 (4.2) | 87 (4.4) | 13 (0.7) |
| **Sepsis**, n(%) | 218 (11.0) | 174 (8.8) | 15 (0.8) |
| **ARDS^d^**, n(%) | 41 (2.1) | 38 (1.9) | 1 (0.1) |
| **Kidney failure**, n(%) | 202 (10.2) | 194 (9.8) | 22 (1.1) |
| **Acute pancreatitis**, n(%) | 17 (0.9) | 21 (1.1) | NA |
| **Respiratory failure**, n(%) | 456 (23.0) | 310 (15.6) | 22 (1.1) |

^a^CHD: Coronary heart disease.

^b^COPD: Chronic obstructive pulmonary disease.

^c^ICH: Intracerebral hemorrhage disease.

^d^ARDS: Acute respiratory distress syndrome.


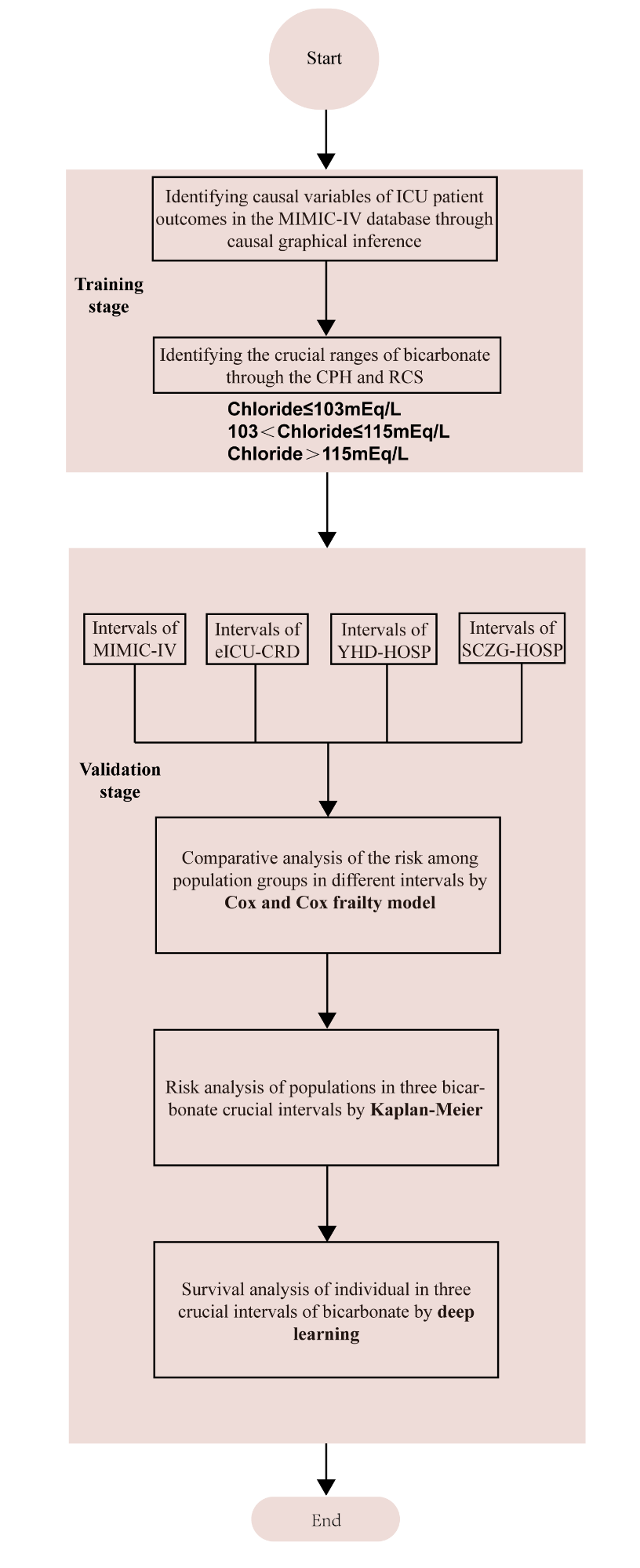


**Figure S1 Analysis flow chart. This figure shows our research process.** MIMIC-IV is the main model study, and eICU, YHD-HOSP and SCZG-HOSP data sets are used as validation, which fully proves the universality and robustness of our study.

The analytical process comprises two main stages:

1. Discovery Phase (MIMIC-IV Database):
   - Employed causal graphical inference, Cox Proportional Hazards (CPH) model, and Restricted Cubic Spline (RCS) curves to investigate quantification intervals of serum bicarbonate in ICU patients.
   - Analyzed hazard ratios and individual survival probabilities for patients within these intervals.
2. Cross-Country Validation Phase (eICU-CRD, YHD-HOSP and SCZG-HOSP Databases):
   - Assessed the generalizability of critical serum chloride values (103 mEq/L and 115 mEq/L) identified in the MIMIC-IV database across multinational cohorts.

Methodology

This study introduced a novel, data-driven approach to explore the relationship between critical serum chloride intervals and in-hospital mortality:

1. Variable Selection:
   - Integrated a data-driven causal inference algorithm with expert knowledge in critical care.
   - Identified 42 covariates as adjustment variables for the final analysis
   - Purpose: Determine the impact of main variables
2. Critical Interval Determination:
   - Utilized the Cox Proportional Hazards model and Restricted Cubic Spline to identify the intersection point where bicarbonate and Hazard Ratio (HR) equaled 1.
   - Delineated and analyzed crucial intervals within all three datasets (MIMIC-IV, eICU-CRD, YHD-HOSP and SCZG-HOSP)
3. Population Stratification:
   - Segregated the study population based on the identified intersection points.
   - Enabled subsequent evaluations of risk associations and changes in survival probability.

This refined approach aims to provide a more robust and comprehensive analysis of the relationship between serum chloride levels and in-hospital mortality in critically ill patients, utilizing both advanced statistical techniques and clinical expertise.


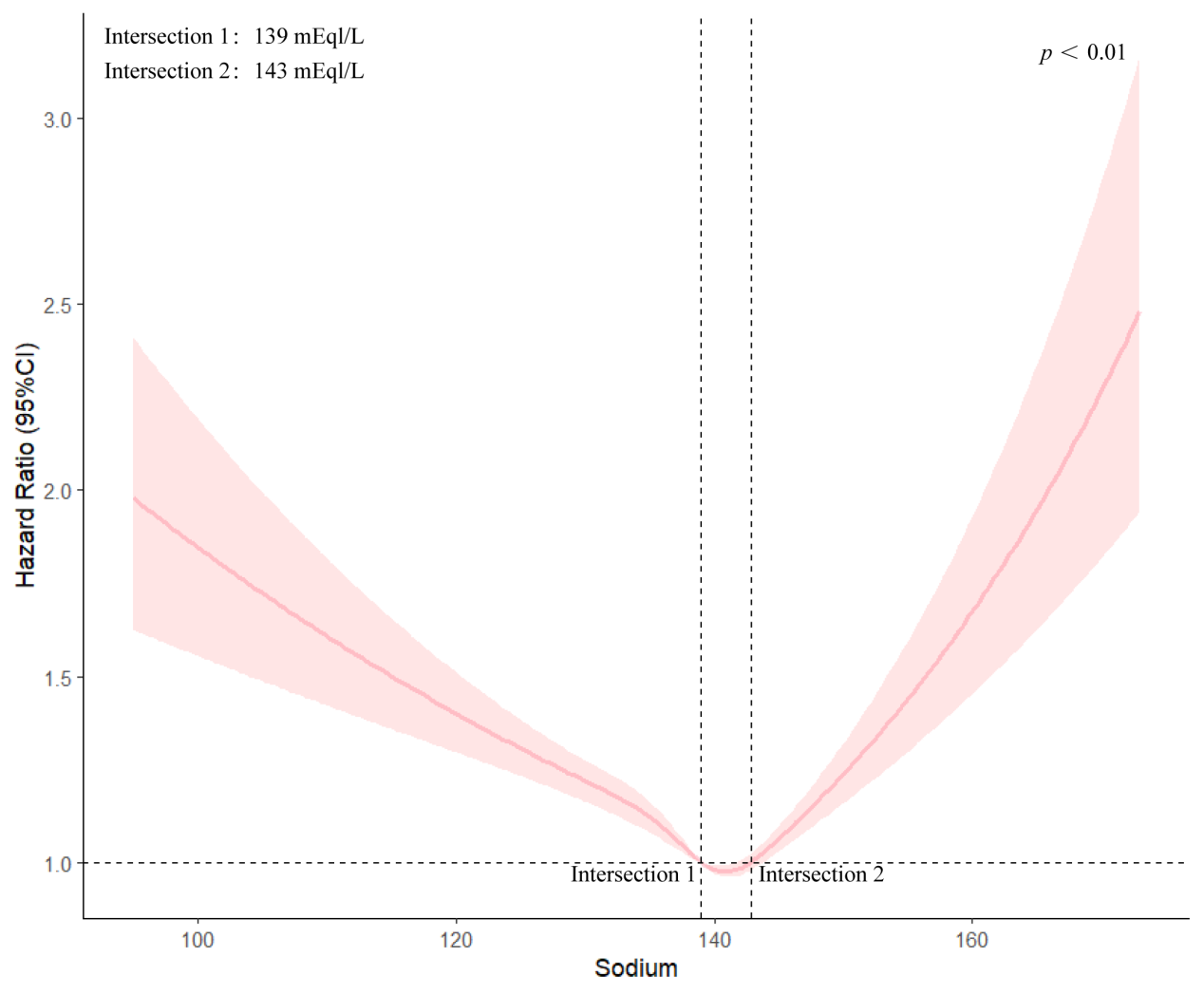


**Figure S2 RCS curve for sodium in the MIMIC cohort.** The Cox model and RCS analysis revealed two inflection points (139 and 143 mEq/L), categorizing sodium into three critical ranges.

**
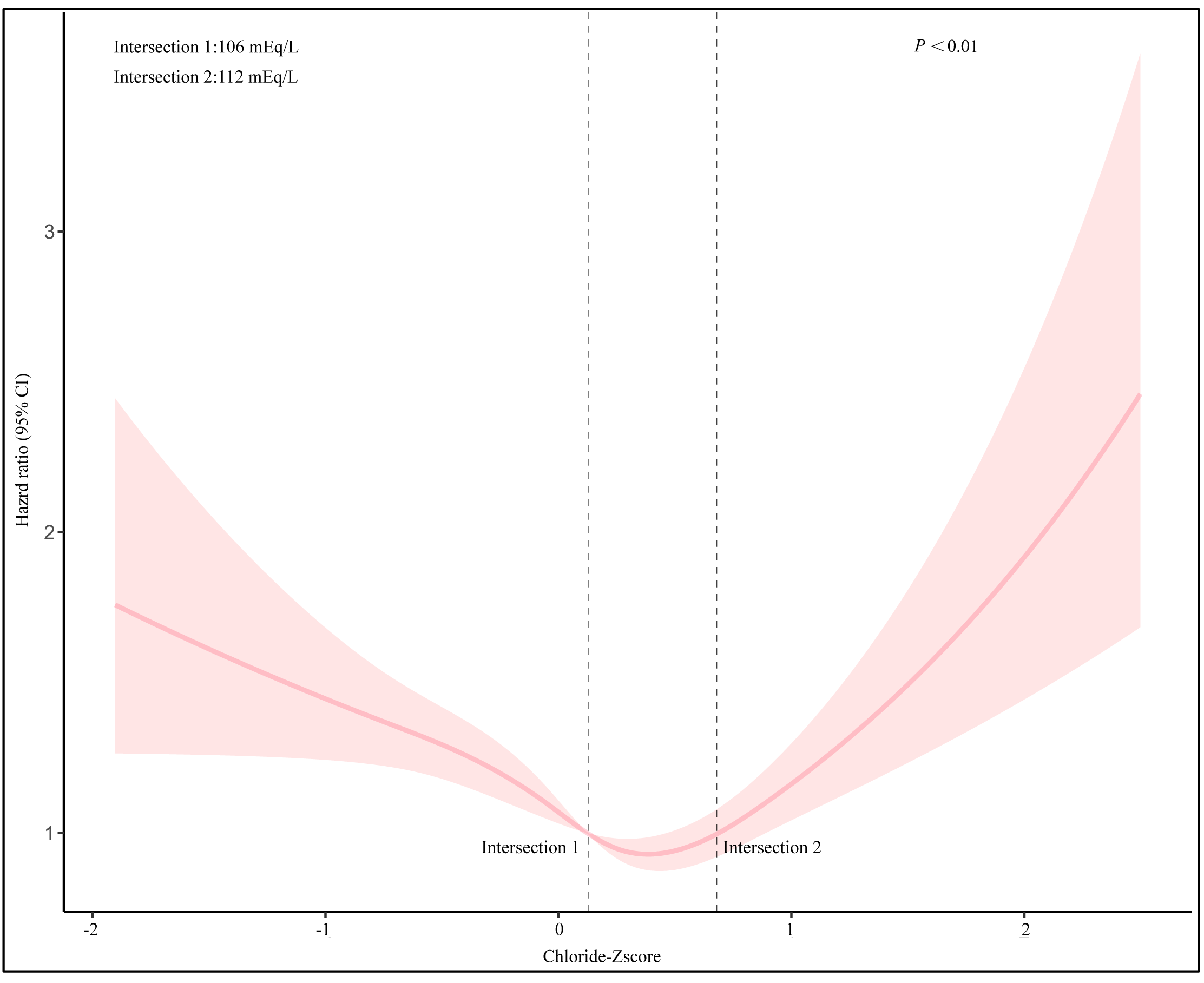
**

**Figure S3 The YHD-HOSP RCS image curve.** This study employs COX proportional hazard models and restricted cubic spline curves to identify two intersections of chloride, dividing them into three key intervals through intersection 1 (106 mEq/L) and intersection 2 (112 mEq/L).


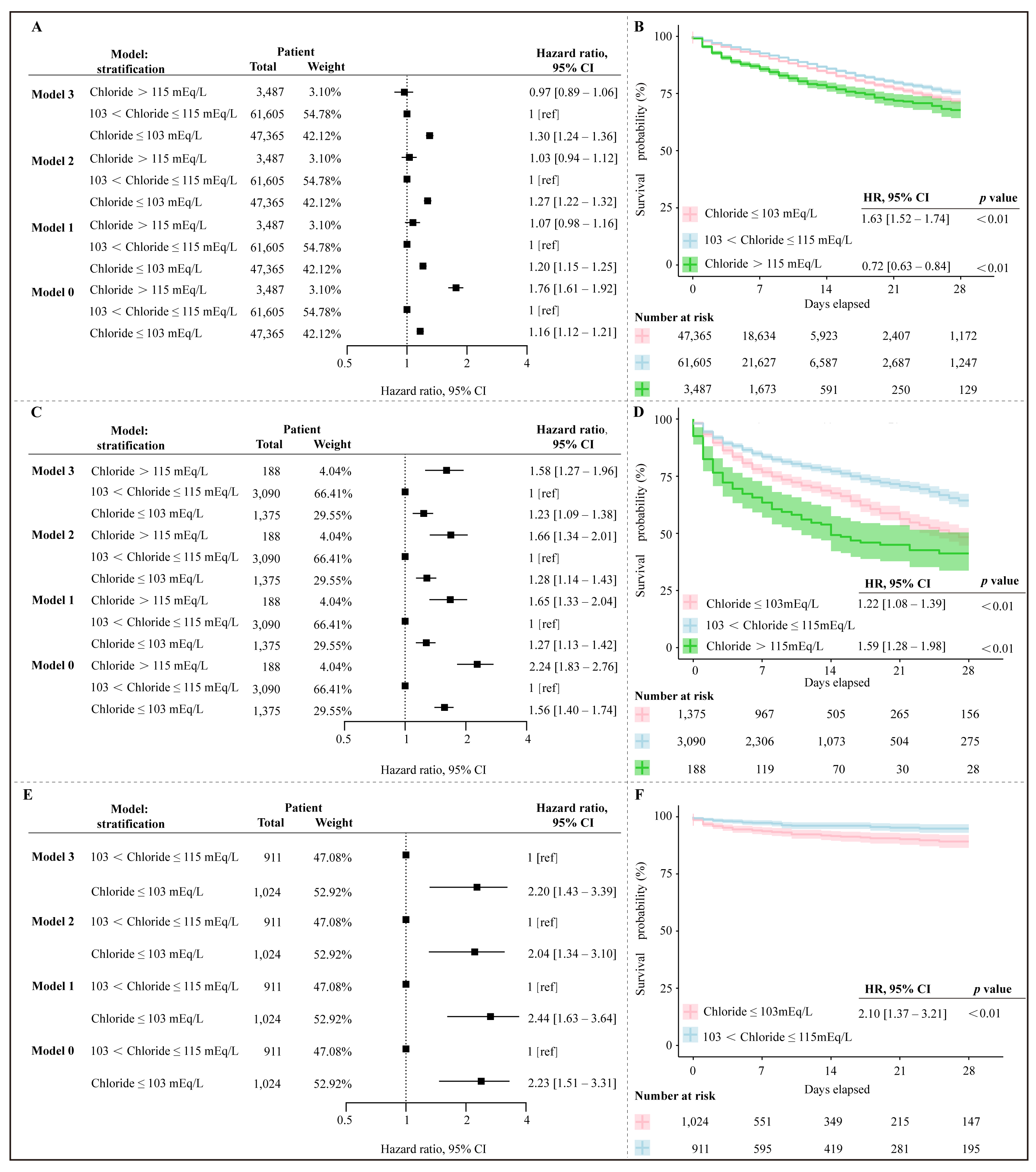


**Figure S4 Cross-cohort and cross-country validation of serum chloride thresholds associated with in-hospital mortality.** (**A**) Cox regression models stratified by serum chloride levels in the eICU-CRD cohort. Compared to the reference range (103-115 mEq/L), patients with chloride levels ≤ 103 mEq/L had an increased hazard ratio (HR) of 1.30 (95% CI 1.24 -1.36). (**B**) Kaplan-Meier survival curves for the eICU-CRD cohort. Patients with chloride levels ≤ 103 mEq/L or > 115 mEq/L exhibited lower survival probabilities. (**C**) Cox regression models stratified by serum chloride levels in the YHD-HOSP cohort. HR for chloride levels ≤ 103 mEq/L was 1.23 (95% CI 1.09 -1.38) and for > 115 mEq/L was 1.58 (95% CI 1.27 -1.96). (**D**) Kaplan-Meier survival curves showing survival probabilities for the YHD-HOSP cohort over 28 days post ICU admission for different admission chloride levels. (**E**) Cox regression models stratified by admission serum chloride levels in the SCZG-HOSP cohort. HR for chloride levels ≤ 103 mEq/L was 2.28 (95% CI 1.36-3.20). **(F**) Kaplan-Meier survival curves depicting survival probabilities for the SCZG-HOSP cohort over 28 days post ICU admission for different admission chloride levels.

Model 0: Including serum chloride and outcome variables.

Model 1: Model 0 + demographics (age, sex, smoking status, marital status, and race) + underlying diseases (chronic obstructive pulmonary disease (COPD), cirrhosis, hepatic failure, fatty liver, malignancy, cerebral infarction, cerebral infarction, coronary heart disease (CHD), solid tumors, cerebral hemorrhage (ICH), hypertension, hyperlipidemia, diabetes, and obesity) + critical illness (sepsis, acute pancreatitis, acute respiratory distress syndrome (ARDS), kidney failure, and respiratory failure).

Model 2: Model 1 + ions and electrolytes in blood (potassium, phosphate, magnesium, and bicarbonate) + sequential organ failure assessment (SOFA)/ acute physiology and chronic health evaluation iv (APACHE IV).

Model 3: Model 2 + biochemical indicators (platelet count, platelet count, white blood cell (WBC) count, creatinine, international normalized ratio (INR), hemoglobin, glucose, and urea nitrogen) + vital signs (peripheral oxygen saturation (SpO2), respiratory rate, mean blood pressure (MBP), heart rate, and temperature).

**
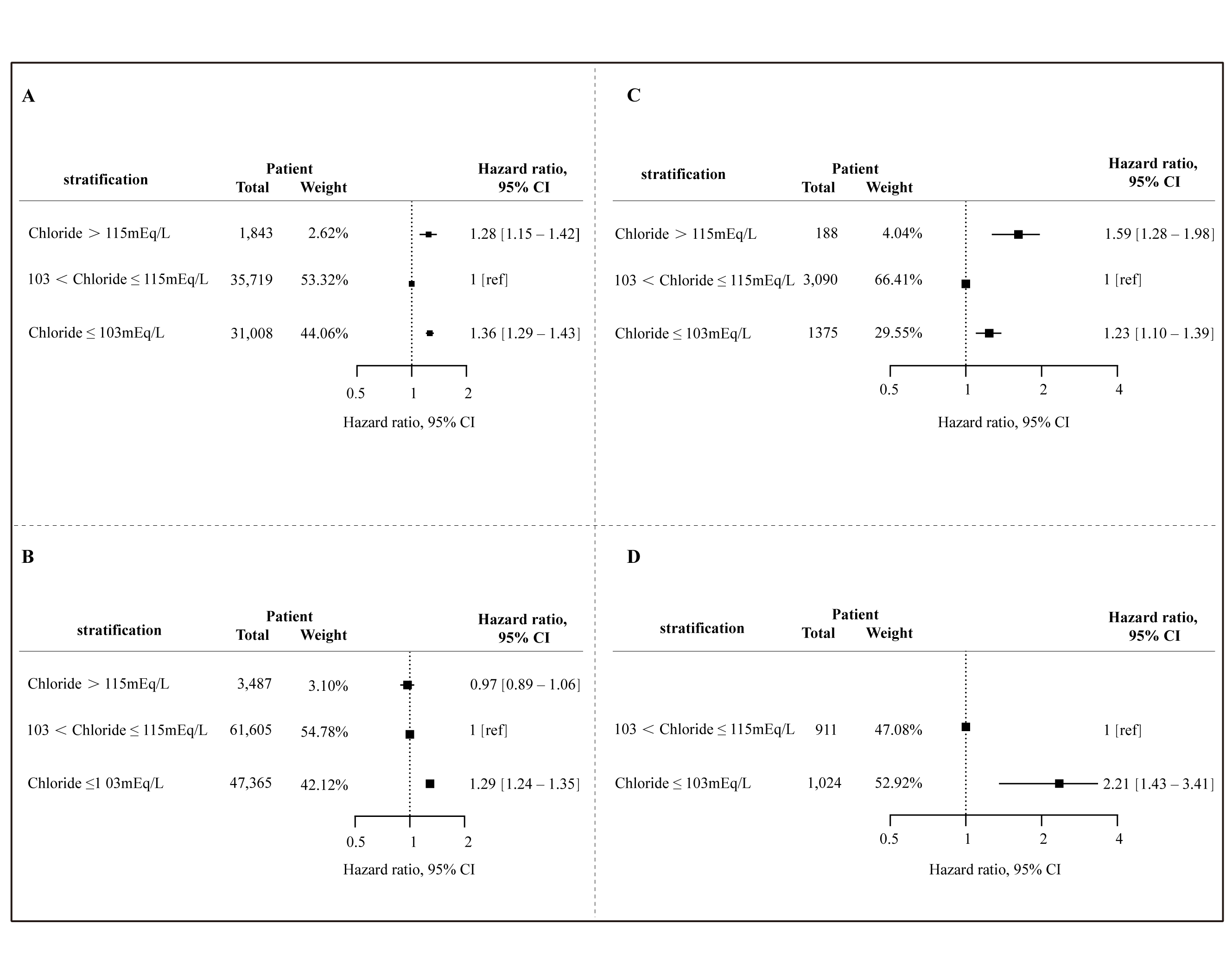
**

**Figure S5 Sensitivity analyses confirming the robustness of the association between serum chloride levels and in-hospital mortality after adjusting for Acute Kidney Injury (AKI).** Sensitivity analyses reveal that the significant associations between serum chloride levels and in-hospital mortality remain robust even after accounting for AKI in the Cox regression models. A. Cox regression analysis for the MIMIC-IV cohort, showing that patients with chloride levels ≤ 103 mEq/L (HR 1.36, 95% CI 1.29 - 1.43) and > 115 mEq/L (HR 1.28, 95% CI 1.15 - 1.42) have increased mortality risk compared to the reference range (103-115 mEq/L). B.Cox regression analysis for the eICU-CRD cohort, indicating that patients with chloride levels ≤ 103 mEq/L (HR 1.29, 95% CI 1.24 - 1.35) have increased mortality risk, whereas those with levels > 115 mEq/L do not show a significant difference (HR 0.97, 95% CI 0.89 - 1.06) compared to the reference range. C. Cox regression analysis for the YHD-HOSP cohort, demonstrating the increased mortality risk for patients with chloride levels ≤ 103 mEq/L (HR 1.23, 95% CI 1.10 - 1.39) and > 115 mEq/L (HR 1.59, 95% CI 1.28 - 1.98) compared to the reference range. D. Cox regression analysis for the SCZG-HOSP cohort, showing a significant increase in mortality risk for patients with chloride levels < 103 mEq/L (HR 2.21, 95% CI 1.43 - 3.41) compared to the reference range. These results confirm that the increased mortality risk associated with serum chloride levels below the lower limit or above the upper limit remains consistent even after adjusting for AKI, supporting the robustness and reliability of the primary findings.

**
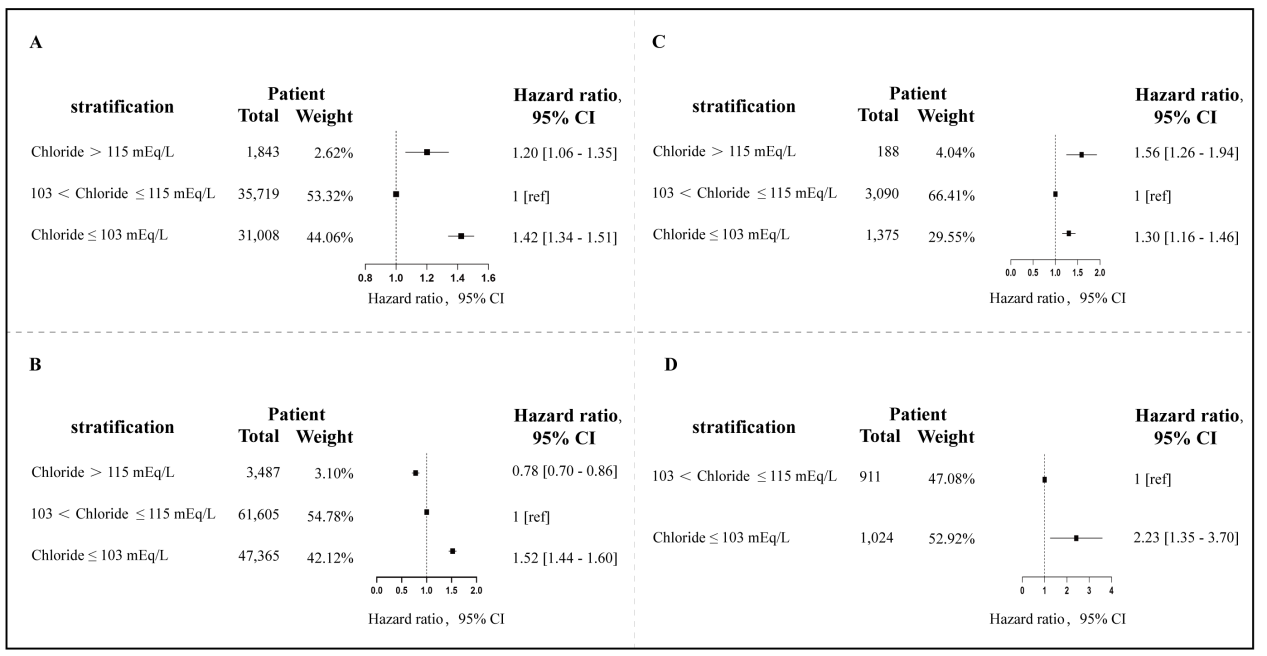
**

**Figure S6 Sensitivity analyses validating the consistency of the relationship between serum chloride concentrations and in-hospital mortality after sodium adjustment.** Sensitivity analyses demonstrate that the significant links between serum chloride levels and in-hospital mortality persist even when sodium is included as a covariate in Cox proportional hazards models. A. Cox regression analysis for the MIMIC-IV cohort reveals that patients with chloride levels ≤ 103 mEq/L (HR 1.42, 95% CI 1.34–1.51) or > 115 mEq/L (HR 1.20, 95% CI 1.06–1.35) exhibit higher mortality risk relative to the reference range (103–115 mEq/L). B. Cox regression analysis for the eICU-CRD cohort indicates that patients with chloride levels ≤ 103 mEq/L (HR 1.53, 95% CI 1.44–1.60) have elevated mortality risk, whereas those with levels > 115 mEq/L show protective significant difference (HR 0.78, 95% CI 0.70–0.86) compared to the reference range. C. Cox regression analysis for the YHD-HOSP cohort confirms an increased mortality risk for patients with chloride levels ≤ 103 mEq/L (HR 1.30, 95% CI 1.16–1.46) or > 115 mEq/L (HR 1.56, 95% CI 1.26–1.94) relative to the reference range. D. Cox regression analysis for the SCZG-HOSP cohort demonstrates a substantially higher mortality risk for patients with chloride levels < 103 mEq/L (HR 2.23, 95% CI 1.35–3.70) compared to the reference range. These findings reinforce that the heightened mortality risk linked to serum chloride levels outside the normal range remains stable after sodium adjustment, underscoring the reliability and generalizability of the primary results.
